# The Gut Microbiome in Early Ontogeny: Implications for Brain and Immune System Development

**DOI:** 10.3390/jdb14020027

**Published:** 2026-06-04

**Authors:** Alejandro Borrego-Ruiz, Juan J. Borrego

**Affiliations:** 1 Departamento de Psicología Social y de las Organizaciones, Universidad Nacional de Educación a Distancia (UNED), 28040 Madrid, Spain; a.borrego@psi.uned.es; 2 Departamento de Microbiología, Universidad de Málaga, 29071 Malaga, Spain

**Keywords:** gut microbiome, brain development, immune system development, neurodevelopmental disorders, early-life mental health

## Abstract

The gut microbiome plays a pivotal role in modulating multiple physiological processes from the earliest stages of life. However, the complete scope of its effects during childhood is yet to be fully elucidated, which underscores the importance of enhancing the understanding of this emerging area of research. This narrative review provides an overview of the influence of the gut microbiome in early human ontogeny by examining its role in brain and immune development, as well as its involvement in neurodevelopmental disorders and early-life mental health. The gut microbiome contributes to shaping the development and function of both the brain and the immune system. Its influence appears to be primarily mediated through the synthesis of neurotransmitters and microbial metabolites, as well as through the activation of specific pathways within the hypothalamic–pituitary–adrenal axis. Nevertheless, the exact mechanisms through which the gut microbiome exerts these effects, and the full extent of its impact on neurodevelopmental and immune health, remain incompletely understood and continue to be active areas of research and scientific debate. Ultimately, advances revealing how the gut microbiome shapes early brain and immune system development will create new opportunities for innovative interventions and predictive strategies aimed at transforming pediatric health outcomes.

## 1. Introduction

The human gut microbiome (GM) consists of a diverse community of microorganisms, including archaea, fungi, protozoa, viruses, and bacteria, which together create a complex and particular ecosystem [1]. While the terms “microbiota” and “microbiome” are often used equivalently, the microbiome is a broader concept that refers to the microorganisms themselves but also to their structural components, collective genomes, derived metabolites, and the surrounding environmental conditions [2]. The development of the GM follows a trajectory that begins in the postnatal period and continues throughout the host’s life, spanning from infancy to old age [1,3]. The initial colonization of the gastrointestinal (GI) tract by GM occurs during fetal development [4]. This process of microbial colonization is closely linked to brain development during pregnancy and remains influential for several years after birth [5]. Over time, the composition of the GM stabilizes, reaching a state of equilibrium that fosters intricate interrelationships between the microorganisms and the human host. However, disruptions in this balance, due to various intrinsic and extrinsic factors, can lead to dysbiosis [6,7]. In the first year of life, the GM exhibits substantial variability between individuals before stabilizing into an adult-like composition, typically around the age of three [8]. This variability can play a pivotal role in brain development and influence the establishment of an individual’s immune system [9,10].

The gut–brain axis (GBA) refers to the connection between the GI system and the brain, enabling bidirectional communication through the endocrine, nervous, and immune systems [11,12]. This interaction allows sensory signals from the gut to be relayed via the vagus nerve, affecting the central nervous system (CNS) by influencing reflexes and regulating mood. In turn, the brain interprets these signals to modulate gut physiology and other organism-related processes. Signal transmission occurs along several pathways, including the sympathoadrenal axis, enteric nervous system (ENS), hypothalamic–pituitary–adrenal (HPA) axis, autonomic nervous system (ANS), and descending monoaminergic routes, utilizing afferent (i.e., that receive) and efferent (i.e., that send) neurons [13]. Each of these pathways is regulated by a variety of neurohumoral and inter-relational factors that add complexity to the system. A pivotal component in this network is the ENS, which is central to the regulation of gut functions. Consisting of the myenteric and submucosal plexuses, the ENS governs key processes such as secretion, absorption, and peristalsis [14]. Within the gut–brain interplay framework, the ENS transmits signals to the CNS via intestinofugal neurons that establish connections with the sympathetic nervous system. Simultaneously, sensory signals travel through vagal afferent fibers [14]. In addition, the CNS can influence the GI system through specific signals that alter its physiological activity. The GM plays a role in this communication by producing metabolites that interact with host cells, which in turn affect synaptic activity in the ANS [15]. Moreover, the ANS can impact the gut epithelium, potentially affecting immune system response. This impact may occur through two main mechanisms: directly modulating immune cell responses to the GM or altering how the GM interacts with immune cells [16].

Prenatal stress, including maternal stress during pregnancy, has been shown to impact fetal brain development and alter the composition of the GM [17,18]. Both preclinical and clinical research suggests that dysbiosis in the GM during perinatal periods might contribute to these effects via the GBA, potentially promoting immune dysfunction, triggering systemic inflammation, and contributing to neurodevelopmental and psychiatric conditions as well as behavioral issues [19,20,21,22,23]. Furthermore, the GBA plays an important role in regulating certain brain functions, including myelination [24], microglial maturation [25,26], and neuronal plasticity [27]. In turn, both prenatal and postnatal stress have been linked to dysregulation of the HPA axis, leading to elevated cortisol levels and alterations in both immune function and GM composition [28,29].

Building on the aforementioned, it can be asserted that the GM might play a pivotal role in shaping and modulating multiple physiological processes from the earliest stages of life. In fact, evidence suggests that early-life GM dysbiosis is linked to several neurodevelopmental disorders (NDDs), such as autism spectrum disorder (ASD) and attention-deficit/hyperactivity disorder (ADHD) [3], as well as to certain neurogenetic conditions, including Turner, Angelman, Down, and Rett syndromes [17]. In addition, GM dysbiosis in early life has been shown to impact immune system maturation, contributing to the onset of metabolic disorders, allergies, atopic diseases, and autoimmune conditions [21]. However, the full extent of its effects during childhood is yet to be fully clarified, which underscores the importance of synthesizing current knowledge and enhancing the understanding of this emerging area of research. Considering its potential involvement in key development-related processes (e.g., regulation of gene expression, production of microbial neuroactive compounds, modulation of microglial activity), the present narrative review provides an overview of the influence of the GM in early human ontogeny by examining its role in (i) brain development and function; (ii) immune system development; (iii) NDDs; and (iv) early-life mental health.

## 2. Method

A narrative, non-systematic review was conducted to examine the role of the GM in early ontogeny, with a focus on a descriptive overview of its implications. Given the wide scope of the topic, this methodological approach was deemed the most appropriate, as it allows for a comprehensive synthesis of findings without the constraints of standard systematic review protocols. Literature searches were performed between December 2025 and February 2026 using PubMed, Scopus, and Web of Science databases. Various keywords related to the topic under investigation were combined iteratively to capture relevant studies. In addition, reference lists of key articles were examined to identify further pertinent literature. No formal restrictions on publication date or language were applied. Following initial screening of titles and abstracts, duplicates and studies deemed irrelevant were excluded. Inclusion criteria focused on studies addressing the potential influence of the GM during early ontogeny, including its role in brain and immune development, NDDs, and early-life mental health. Exclusion criteria encompassed research exclusively focused on unrelated topics, such as adult-onset conditions or findings outside the scope of neurodevelopmental or immunological relevance. Both human and animal studies were considered to provide mechanistic insights. Given the narrative nature of this review, a quality assessment of the included studies was not performed. Relevant findings were organized into thematic sections, emphasizing descriptive patterns of GM influence primarily across developmental and immunological domains.

## 3. Microbial Colonization During Human Embryonic Development

Until the early 2000s, the neonatal gut was widely considered a sterile environment, a concept commonly referred to as the “sterile womb paradigm” [30], with microbial colonization thought to begin only at birth through vertical transmission from the maternal microbiota and horizontal acquisition from the environment [3,8]. Emerging evidence has challenged this view, revealing the presence of bacterial cells or microbial DNA in the amniotic fluid, meconium, placenta, and umbilical cord blood of healthy infants, including those delivered by C-section [8,31,32]. This has led to the formulation of the in utero colonization hypothesis, which has been supported by research detecting probiotics ingested by pregnant women in both the placenta and the meconium of full-term newborns [33,34]. Throughout gestation, maternal endocrine, immunological, and metabolic adaptations create an intrauterine milieu that promotes optimal fetal development [35]. These physiological changes are associated with a pro-inflammatory state that induces shifts in the maternal vaginal, gut, skin, and oral microbiomes [36]. Importantly, maternal microbial transfer to the fetus is thought to contribute significantly to the establishment of the neonatal microbiome, potentially shaping early immune system maturation [37] and influencing nervous system development [38].

Traditionally, the uterus has been regarded as a sterile organ in the absence of infection. Nevertheless, the microbial composition of endometrial tissue and mucus obtained from 19 non-pregnant women undergoing hysteroscopy, all without uterine abnormalities, was characterized using 16S rRNA gene profiling [39]. Remarkably, bacteria were detected in every sample, providing evidence for the existence of a natural microbiota within these uterine compartments [39]. Several hypotheses have been proposed to explain how bacteria may colonize the uterine cavity during a healthy pregnancy [40], including: (i) vertical ascent from the vaginal canal and/or urinary tract, and (ii) hematogenous transmission via the placenta following microbial translocation from the GI tract or oral cavity [40].

In recent years, the traditional view of in utero sterility has been increasingly questioned. Accumulating evidence now indicates the presence of bacteria or bacterial DNA within healthy placental tissues [40,41,42]. Moreover, the assumption that fetuses develop in a sterile environment has been further challenged by the detection of commensal bacteria in meconium from neonates delivered via both vaginal birth and C-section [43]. These observations suggest that full-term fetuses may harbor microbial communities, pointing to the possibility of maternal–fetal transfer of commensal bacteria across the placenta. Supporting this, bacterial species including *Enterococcus faecium*, *Cutibacterium* (formerly *Propionibacterium*) acnes, *Staphylococcus epidermidis*, and *Streptococcus sanguinis* have been isolated from umbilical cord blood in healthy neonates delivered by C-section [44].

A microbiological analysis detected intracellular bacteria of varied morphologies within the maternal basal plate in 27% of 195 placentas examined, although no taxonomic identification was reported, while no significant differences were observed between basal plates from preterm and term gestations [45]. In addition, intracellular bacteria have been found in placentas without clinical or pathological signs of chorioamnionitis [46]. The use of whole-genome shotgun metagenomics on placental samples collected under aseptic conditions from 320 subjects revealed that the placenta hosts a low-abundance but metabolically active, site-specific microbiome, primarily composed of nonpathogenic commensals belonging to the Bacillota, Mollicutes, Pseudobacteriodota, Bacteroidota, and Fusobacteriota phyla [47]. At the species level, *Escherichia coli* appears to dominate placental bacterial communities. Interestingly, the placental microbial profiles and their genomic characteristics show notable similarities to those found in the oral cavity [48]. Moreover, the predominance of *E. coli* suggests a direct or indirect link between the placental microbiota and the maternal GM, where this species is highly prevalent [49]. Thus, these observations indicate that the maternal digestive tract, spanning from the oral cavity to the distal colon, may play a central role in placental microbial colonization.

Amniotic fluid encloses the fetus and is continuously ingested during gestation. Bacteria are present in fetal compartments including amniotic fluid, even without signs of infection, often matching species found in cord blood [43,44]. A recent study using 16S rRNA gene profiling found that within mother-infant pairs, the bacterial communities in meconium closely resemble those of the maternal placenta, regardless of delivery mode, although they remain distinct from the maternal vaginal microbiota [50]. Since meconium harbors a complex microbial community similar to that of the placenta, research has examined its microbial diversity [51,52,53]. A study on the first-pass meconium from 15 healthy full-term infants delivered vaginally found that approximately 66% of the neonates harbored viable bacteria [51]. The study identified a diverse range of microbial groups, with *Bifidobacterium*, members of the *Enterobacteriaceae* and *Enterococcaceae* families, and the *Bacteroides*–*Prevotella* group being the most prevalent. These findings suggest that, although bacteria are present in the meconium of healthy, vaginally delivered, breastfed term infants, their abundance is relatively low. Additional studies have detected bacterial DNA from a variety of species in the meconium of healthy newborns [4,53,54], providing further support for the concept that gut microbial colonization begins before birth.

In summary, the detection of microorganisms in the healthy human placenta, umbilical cord, and meconium indicates that fetal microbial exposure may represent a pivotal physiological process. However, the precise role of intrauterine microorganisms in shaping infant gut colonization remains unclear. The observation of distinct microbial communities in the placenta, amniotic fluid, and first-pass meconium among individuals suggests that active and selective mechanisms may facilitate the transfer of bacteria to these maternal and fetal compartments. Building on these insights, it is plausible that the maternal-fetal microbiome, far from being a mere passive reservoir, functions as an evolutionarily shaped interface, potentially “programming” neonatal immune and metabolic systems while guiding early adaptability and neurodevelopmental trajectories.

## 4. Gut–Brain Axis Connections

The signaling pathways that connect the gut and brain within the GBA are bidirectional. The brain substantially impacts GI activity through the ANS and the HPA axis. For instance, when norepinephrine is released by the brain in response to stress, it promotes the growth of various microbial species in the GM [55]. In turn, the gut may also play a role in shaping CNS activity by transmitting gut hormones, neuroactive compounds, and microbial metabolites. These signals travel through pathways such as the circulatory system, the vagus nerve, the immune system, or the ENS [56]. Together, these microbial byproducts may contribute to establishing a signaling network that can directly influence brain health and function.

Short-chain fatty acids (SCFAs) are produced by microbial fermentation of carbohydrates and are essential for maintaining gut integrity [57]. SCFAs can cross the blood–brain barrier (BBB) and interact with G-protein coupled receptors (GPRs) such as GPR41 and GPR43, which help regulate neuroinflammation and neurotransmitter release [58]. Similarly, neurotransmitters such as glutamate, dopamine, serotonin (5-HT), and gamma-aminobutyric acid (GABA) constitute pivotal mediators in the GBA, influencing both peripheral and central processes [59]. For instance, enterochromaffin cells produce 5-HT, which regulates GI motility and transmits signals to the CNS via the vagus nerve [59].

The vagus nerve plays a pivotal role as the primary route associating the gut with the brain, making it a particularly important component of the gut–brain communication network [60]. Recent research has highlighted a strong connection between the vagus nerve and the CNS, especially in relation to its influence on reward neurons. This interaction has been shown to affect mood regulation and behavior within the CNS [61]. The GM is essential for initiating, regulating, and maintaining both systemic and local immune responses, encompassing both innate and adaptive immunity [62]. This emphasizes the potential role of the gut-immune-brain axis in sustaining overall health. In this context, preclinical studies have demonstrated that germ-free (GF) mice experience significant immune deficiencies, such as increased susceptibility to infections, and these deficiencies have been attributed to factors such as a lack of the mucous layer, altered IgA secretion, diminished Peyer’s patch dimension and function, and a decrease in pro-inflammatory T-helper (Th) cell activity [63].

## 5. The Gut Microbiome in Brain Development

The hypothesis that the GM influences brain development was first proposed in 2011, when it was observed that GF mice exhibited altered motor activity and reduced anxiety-like behavior [64]. This hypothesis was later supported by evidence showing that the probiotic *Lacticaseibacillus* (formerly *Lactobacillus*) *rhamnosus* strain JB-1 displayed anxiolytic and antidepressant effects [65]. In addition, GF animals showed impairments in memory and locomotor tasks [66], a reduction in adult hippocampal neurogenesis [67], and diminished long-term enhancement of the cornu ammonis 1 (CA1) area within the hippocampus [68]. Research has also demonstrated that antibiotic treatment can alter the GM and induce behavioral deficits by diminishing adult hippocampal neurogenesis and affecting synaptic transmission [69]. Notably, fecal microbiota transplantation (FMT) from young donor mice has been shown to reverse age-related cognitive decline [70]. Furthermore, reversal experiments in young rats led to cognitive and behavioral deficits, as well as a decrease in dendritic spines within the hippocampus and prefrontal cortex (PFC) [71]. Various mechanisms have been suggested in order to explain how alterations in the GM could affect brain plasticity. These may include the production of microbial neuroactive substances (e.g., metabolites and neurotransmitters) and the regulation of gene expression [27].

### 5.1. Gene Regulation

The GM generates various metabolites that can travel through the bloodstream to distant tissues, including the CNS. These metabolites have the potential to impact epigenetic marks [72]. Epigenetic alterations, including histone acetylation, DNA methylation, microRNA-mediated transcriptional silencing, and the regulation of long noncoding RNAs, have all been observed to influence the brain’s plasticity processes [73].

Epigenetic regulation is controlled by specific enzymes, the activity of which is influenced by microbial metabolites [74]. SCFAs have been shown to promote histone acetylation, facilitating the binding of transcription factors to DNA and, as a result, enhancing gene transcription [75]. Another important area of research is the role of SCFAs in histone crotonylation, a form of post-translational modification [76,77]. Moreover, SCFAs have been found to induce plasticity in the visual cortex of adult mice. This effect is linked to changes in microglial morphology [78], and also to alterations in gene expression within primary cortical astrocytes [79].

As previously noted, another key epigenetic modification implicated in neuronal plasticity and linked to the GM is host DNA methylation. Indeed, changes in DNA methylation have been observed during processes of learning and the consolidation of memory. Research has highlighted the role of DNA methylation in experience-dependent plasticity and the regulation of synaptic function [27,80]. DNA methylation is affected by the one-carbon metabolism route, which depends on the availability of cofactors that constitute a requisite for DNA methyltransferases [81]. Several of these cofactors are microbial metabolites, including folate, riboflavin, cobalamin, and pyridoxine [82,83]. Although the GM appear to induce DNA methylation alteration within gut epithelial cells [84], there is currently no evidence suggesting that the GM directly affects DNA methylation or transcription in the brain. Furthermore, there is no supporting evidence linking these changes to alterations in network plasticity [27].

MicroRNAs (miRNAs) constitute small noncoding RNAs that play a pivotal role in posttranscriptional gene silencing. This process occurs through the binding of miRNAs to target mRNAs, leading to the inhibition of their expression and function across various systems, including the CNS [85]. Research has shown that the GM may influence the expression of miRNAs, which in turn regulate various brain functions. These include spine density in neurons of the hippocampus, dendritic morphology, control of visual cortical plasticity by modulating spine remodeling, and overall regulation of cortical plasticity [86,87,88]. Preclinical research has also suggested that the absence of intestinal microbiota may affect miRNA expression, particularly in brain regions such as the amygdala and PFC, which are implicated in the regulation of anxiety and fear responses [89,90].

### 5.2. Neuroactive Molecules

#### 5.2.1. Metabolites Derived from the Gut Microbiome

Microbial metabolites produced by the GM, such as SCFAs and tryptophan-derived compounds, have been shown to influence various host cells and tissues. SCFAs, in particular, are thought to support various physiological and mental processes, including mucosal secretion, lymphocyte function, glucose homeostasis, as well as learning and memory, all through the maintenance of BBB integrity [63]. In addition, these metabolites regulate epithelial barrier function as well as mucosal and systemic immunity. The mechanisms underlying these effects involve evolutionarily conserved pathways, including GPR signaling and histone deacetylase activity [58]. Butyrate, one of the SCFAs, plays a key anti-inflammatory role by directly influencing the differentiation of phagocytes, intestinal epithelial cells, B cells, plasma cells, and both regulatory and effector T cells. Moreover, SCFAs derived from the intestine have been shown to affect immune responses at extraluminal sites, such as the lungs, liver, and brain. These metabolites have been related to various conditions, such as intestinal inflammation, infections, food allergies, autoimmune disorders, asthma, and responses to cancer therapies [91].

Beyond their local effects in the colon and peripheral tissues, SCFAs are considered to play an important role in mediating communication within the GBA. The high expression of H^+^-dependent and sodium-dependent monocarboxylate transporters in endothelial cells may facilitate the crossing of SCFAs through the BBB [92]. Growing evidence suggests that SCFAs that pass the BBB exhibit neuroactive properties. Although the exact mechanisms through which SCFAs affect the CNS remain incompletely understood, preclinical studies have provided substantial evidence of their significant impact on key neurological and behavioral processes [93]. Research indicates that SCFAs interact with GPRs, such as GPR41 in enteric neurons and GPR43 in adipose tissue, to mediate various functions [33]. For instance, acetate, which constitutes a major SCFA produced by the GM, has been noted to regulate food consumption in one study [94], while in another, it was inferred to stimulate food intake through ghrelin secretion [95].

Several studies have shown that sodium butyrate can decrease the activation of microglia and also the release of pro-inflammatory cytokines [96,97,98]. Moreover, butyrate treatment, both in vitro and in vivo, has been found to induce morphological and functional changes in microglia, promoting a homeostatic profile. This treatment also inhibits lipopolysaccharide (LPS)-induced pro-inflammatory alterations [97] and alleviates depression-like behavior [98]. In addition, acetate has been shown to attenuate inflammatory signaling by reducing the expression of IL-1β, IL-6, and TNF-α, as well as decreasing phosphorylation of p38 MAPK, JNK, and NF-κB in microglia [99,100]. Although the signaling mechanisms underlying the effects of SCFAs on microglia are becoming clearer, the exact processes remain to be fully understood. One proposed mechanism is the inhibition of histone deacetylases (HDACs), which leads to epigenetically regulated gene expression and is thought to be the primary action triggered by SCFAs [101,102]. Through histone acetylation, SCFAs appear to modulate glial cells in a manner that is both anti-inflammatory and neuroprotective [103]. Given the well-established role of microglia in shaping neuronal networks, combined with the influence of the GM on this process, SCFAs may offer new strategies to modulate brain immune disruption associated with neurodevelopmental and neurodegenerative disorders. Furthermore, SCFAs have been shown to influence neuronal function by regulating neurotransmitter levels and neurotrophic factors [92]. In addition to providing energy to cells, SCFAs impact microglial maturation, with acetate, for instance, altering the levels of neurotransmitters such as glutamine, glutamate, and GABA in the hypothalamus, as well as enhancing the expression of anorexigenic neuropeptides [94].

An important aspect to consider is the conversion of dietary tryptophan into indole compounds by gut microorganisms. Recent findings have shown that certain bacteria, particularly those in the *Lactobacillaceae* family, are essential for activating the aryl hydrocarbon receptor (AHR), which plays a pivotal role in regulating the cell cycle and promoting T-cell differentiation [104]. Research has also demonstrated that tryptophan derived from diet is important for modulating encephalitogenic T-cell responses, which are involved in driving CNS autoimmunity [105]. Primarily derived from the diet, tryptophan constitutes an essential amino acid that is especially important during pregnancy. As the exclusive precursor for 5-HT, maternal circulating tryptophan contributes to fetal brain development both directly and indirectly via the serotonergic pathway. 5-HT is transported to the fetal brain, where it is synthesized locally by tryptophan hydroxylase type 2 (TPH2). In addition, 5-HT is produced at the placenta through TPH type 1 (TPH1) and subsequently delivered to the fetal brain [106]. The majority of 5-HT synthesis (90%) occurs in the distal GI tract, with the remaining 10% produced in the CNS and liver [107]. TPH1 is expressed in enterochromaffin cells of the adipose tissue, GI tract, and pancreatic β-cells, whereas TPH2 is found in serotonergic neurons within both the CNS and the ENS [108]. Tryptophan can also undergo oxidative degradation through the kynurenine (KYN) pathway, producing kynurenines, which are known to suppress T-cell responses [109]. In the human placenta, tryptophan may be metabolized into 5-HT, processed through the KYN pathway, or transported to the developing embryo. As a result, the fetus is exposed to tryptophan and its metabolites (i.e., 5-HT and kynurenines), which together influence neurogenesis, neuroendocrine development, and the composition and diversity of the offspring’s GM [110]. The serotonergic pathway utilizes about 5% of total tryptophan, primarily in locations such as the CNS, the GI tract, adipose tissue, and pancreatic β-cells. This pathway plays a pivotal role in modulating physiological responses to environmental stimuli, including overall cognition, feeding behavior, and sleep [111].

#### 5.2.2. Gut Neurotransmitters

It has been shown that several bacterial taxa within the GM are capable of synthesizing essential neurotransmitters for the CNS, including GABA, 5-HT, norepinephrine, and dopamine [112]. However, certain neurotransmitters, such as dopamine and 5-HT, are not capable of crossing the BBB and must instead be synthesized in the brain from their precursors (i.e., 5-hydroxytryptophan, tyrosine, phenylalanine, and tryptophan). The ability of the GM to produce these precursor molecules, which can reach the brain, suggests that the GM may play a role in shaping cognitive function and behavior by influencing neuronal plasticity [93,112]. Table 1 outlines the primary bacterial genera involved in neurotransmitter synthesis and their effects on the CNS.

5-HT has been shown to regulate a wide range of functions in neurons, including appetite, mood, memory, learning, social behavior, and sleep [120]. The synthesis of 5-HT is primarily catalyzed by enterochromaffin cells in the GI tract, with tryptophan serving as the key precursor, as previously noted. However, existing evidence suggests that the GM may also influence the synthesis of 5-HT within the CNS [121,122]. Moreover, serotonergic signaling plays an important role in synaptic plasticity, affecting processes such as long-term potentiation and depression, which are pivotal for the consolidation of learning and memory [123]. One study even proposed that 5-HT is involved in regulating synaptic plasticity in the PFC during postnatal development [124]. In a significant finding, Yaghoubfar et al. [125] demonstrated that administering *Akkermansia muciniphila*-derived extracellular vesicles increased 5-HT levels in the hippocampus of mice. This increase in 5-HT was associated with a regulatory effect on the expression of genes implicated in the biosynthesis of 5-HT within the brain. Notably, extracellular vesicles produced by bacteria have the potential to enter the bloodstream and penetrate the BBB, suggesting that these vesicles may directly influence other regulatory pathways within the CNS [126].

GABAergic transmission in the CNS plays an important role in regulating both developmental and mature cortical plasticity [127]. Although certain groups of gut microorganisms have been shown to produce GABA [116], there is currently no definitive evidence linking microbial-derived GABA directly to brain plasticity [27]. However, bacterial-derived GABA may influence brain function indirectly by acting locally on the ENS or via the vagus nerve [128]. The probiotic *L. rhamnosus* has been shown to affect the expression of GABA receptors in specific subcortical regions of the brain, such as the locus coeruleus, hippocampus, and amygdala, as well as in various cortical areas, including the prelimbic and cingulate cortices [65]. These alterations were related to reduced anxiety and depressive-like behaviors, enhanced memory consolidation in a fear-conditioning paradigm, and overall modulation of cognitive and emotional processes through GABAergic transmission [129].

Dopamine constitutes a pivotal catecholamine neurotransmitter in mammals that has a significant impact on both the CNS and peripheral nervous system [130]. In the PFC, dopamine plays essential roles in facilitating the transmission of motor commands, sustaining and manipulating working memory, and regulating emotional processing [118]. In addition to its recognized role in reward-related and motivation processes, dopamine systems also influence brain areas implicated in chronic pain [117]. Dopamine synthesized by the GM is processed and subsequently delivered through the BBB, exerting inhibitory effects on the NLRP3 inflammasome via dopamine receptors that are present on both astrocytes and microglia, thus influencing neuroinflammatory activity [131]. The D4 dopamine receptor has been shown to regulate the transport of α-amino-3-hydroxy-5-methyl-4-isoxazole-propionic acid receptors within GABAergic interneurons of the PFC via a unique signaling route. This modulation affects the robustness of excitatory synapses, with significant implications for cognition and emotional regulation [132].

Norepinephrine is another key catecholamine primarily synthesized by noradrenergic neurons within the locus coeruleus. Beyond its neuronal synthesis, norepinephrine is also synthesized by specific gut microorganisms, such as *Proteus vulgaris*, *E. coli*, and *Bacillus subtilis* [119,133]. Research has shown that norepinephrine plays a pivotal role in modulating synaptic plasticity [134], enhancing the production of brain-derived neurotrophic factor (BDNF), as well as regulating the activation of microglia and astrocytes, thereby providing neuroprotective effects [135]. These actions are essential for cognitive functions, including cognitive flexibility and working memory processes [136].

### 5.3. The Gut Microbiome in Early Neurodevelopment and Brain Function

**Figure 1:**
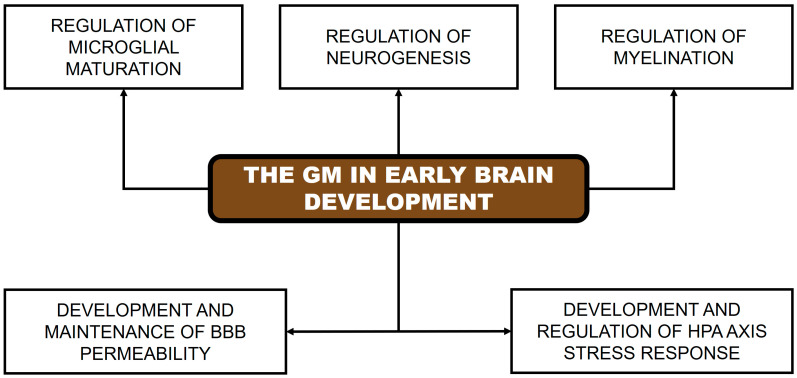
The GM in initial phases of brain development. GM: gut microbiome. BBB: blood–brain barrier. HPA: hypothalamic–pituitary–adrenal.

A balanced GM seems to be essential for supporting optimal brain function and emotional regulation, while the CNS exerts a major regulatory influence over GI physiology. Disruptions in this bidirectional communication have been linked to the development of both neurological and GI disorders [5]. Extensive evidence demonstrates that the GM influences multiple neurodevelopmental processes, including those related to neurogenesis [137], myelination [138], BBB formation [139], microglial maturation [26], and the HPA axis [140], all of which play pivotal roles in shaping child cognition and behavior. Proper maturation and functioning of neuronal cells depend on dietary molecules and gut-derived metabolites [141], and gut microorganisms may directly facilitate neurodevelopment with lasting health implications [142]. The composition of the GM, which is, as previously noted, most variable during the first year of life and typically stabilizes by around three years of age [10], significantly influences both immune profiles and brain developmental processes. Early colonization of mucosal surfaces is therefore crucial for immune system maturation [10], and dysregulation of the GM may lead to systemic inflammation and aberrant brain development and activity, potentially contributing to symptoms associated with NDDs [5,142]. Accordingly, maintaining a balanced GM is fundamental for proper immune function, which in turn supports healthy neurodevelopmental trajectories [10,143]. Figure 1 illustrates the role of the GM in early brain development (adapted from Dash et al. [144]).

#### 5.3.1. Neurogenesis

The process referred to as “neurogenesis” encompasses the differentiation of neural stem and progenitor cells into functional neurons [145]. This mechanism is essential for a range of cognitive processes, including memory formation, learning, and the regulation of stress responses, with the hippocampus acting as a central hub for these functions [146]. The GM contributes, either directly or indirectly, to sustaining the environment required for proper neuronal development [147]. In pediatric animal models of inflammatory bowel disease (IBD), Salvo et al. [148] reported that GM dysbiosis is associated with impaired behavioral outcomes, reduced neurogenesis, heightened neuroinflammation, and altered hippocampal expression of pseudo-response regulators (PRR). Furthermore, preclinical investigations comparing gut-derived metabolites in GF versus specific pathogen-free (SPF) mouse dams have identified numerous compounds capable of modulating prenatal development and crossing the placental barrier to reach the fetal compartment [149].

Research has also shown that bacterial peptidoglycan can cross the placental barrier and enter the fetal brain, where it activates toll-like receptor 2 (TLR2). This activation has been demonstrated to influence the transcription factor forkhead box G1 (FOXG1), which is essential for regulating neurogenesis and overall brain development, ultimately promoting neuronal proliferation in the forebrain [150]. Neuronal plasticity, which refers to the capacity of neurons to remodel their synaptic connections in response to experiences, is closely associated with synapse formation and maturation. Experimental administration of neonatal prebiotics to 22-day-old rats, compared to other types of prebiotics, has been found to elevate hippocampal levels of synaptophysin and BDNF [151]. Moreover, the GM may indirectly shape neuronal plasticity by affecting the migration and maturation of neurons within the CNS. This influence may involve the modulation of the reelin and ephrin B signaling pathways, where reelin (i.e., a membrane glycoprotein that is pivotal for neuronal migration) and ephrin B work together to support the integrity of the gut epithelial barrier [152,153].

#### 5.3.2. Myelination

A well-balanced GM has been identified as a key regulator of myelination. At birth, human CNS axons are largely unmyelinated. Following birth, oligodendrocytes progressively myelinate developing axons by wrapping them through a process that exhibits variability in both the rate of myelination and myelin thickness, continuing into early adulthood [154,155]. Cognitive function is closely dependent on proper myelination, and both neuronal activity and plasticity have been correlated with the degree of myelin coverage [154,156]. The GM modulates this pivotal process by influencing the expression of genes associated with myelination in oligodendrocytes. Disruptions in myelin integrity can negatively affect behavior and overall brain function [157].

Notably, myelination in the PFC occurs later during early development, making this region particularly vulnerable to external perturbations, including GM dysbiosis. For instance, GF mice exhibit abnormal myelin development in the PFC, which correlates with deficits in social behavior [158,159]. In addition, bacterial metabolites such as SCFAs have been shown to positively regulate myelination, ameliorate intestinal barrier dysfunction, and reduce stress-related behavioral abnormalities [158,160]. Oral supplementation with the SCFA butyrate in antibiotic-treated mice restored normal intestinal physiology, reversed behavioral deficits, and corrected myelination impairments, highlighting the pivotal role of the GM in establishing and maintaining the GBA through regulation of PFC myelination [161]. In consequence, these observations underscore the importance of a balanced GM in supporting proper myelin formation and preserving the functional flexibility of the myelin sheath.

#### 5.3.3. Hypothalamus–Pituitary–Adrenal-Axis

The HPA axis describes the endocrine-neurocrine network connecting the hypothalamus, pituitary gland, and adrenal glands in response to stress. Corticotropin-releasing factor (CRF) is a central mediator in this pathway, triggering a cascade of events that culminates in glucocorticoid release from the adrenal cortex, thereby modulating HPA axis activity [162]. Emerging evidence indicates that the GM plays a pivotal role in the maturation and regulation of the HPA axis [163]. For instance, GF mice show elevated CRF mRNA expression in the hypothalamus relative to SPF controls, reflecting an exaggerated stress response mediated by the HPA axis [163]. Furthermore, administration of a probiotic combination containing *Lactobacillus helveticus* and *Bifidobacterium longum* has been reported to significantly reduce anxiety-like behaviors [164].

#### 5.3.4. The Blood–Brain Barrier

The BBB, which serves as a selective interface between the CNS and systemic circulation, is formed early in gestation by capillary endothelial cells and is further supported by tight junction proteins, astrocytes, and pericytes [165]. This structure ensures the regulated transport of essential nutrients and molecules necessary for proper brain function [166]. The development and maintenance of an intact BBB are influenced by a balanced GM and its metabolites, including SCFAs [166,167,168]. Research has shown that BBB permeability is increased in GF mice, a phenomenon linked to decreased expression of key junctional proteins such as claudin-5 and occludin within the brain endothelium [169]. Importantly, this heightened permeability can be mitigated either by colonizing the gut with a normal microbial community or through administration of butyrate, underscoring the pivotal role of the GM in BBB integrity [167].

#### 5.3.5. Microglial Maturation

Microglial cells constitute the brain’s exclusive resident immune cells, specialized in responding to a wide variety of immune signals and in regulating CNS plasticity, as well as processes related to learning and memory [170,171]. During brain development, microglia are pivotal in both the elimination and formation of synapses. This function is facilitated by direct interactions with synaptic structures and the secretion of diffusible factors [172,173,174,175]. In adulthood, microglia continue to influence synaptic plasticity driven by experience [176], and several cognitive functions [175,177]. Erny et al. [26] were among the first to show that the GM plays a pivotal role in shaping the microglial phenotype. In the absence of a GM, microglia showed a non-mature profile and altered morphology. However, these changes were reversible when microbial SCFAs were introduced. Moreover, a prolonged absence of the maternal microbiome has been shown to induce microglial changes during the prenatal stage [178,179]. In this context, acetate has been found to be central for microglial maturation, thereby regulating their homeostatic metabolic state [180].

Preclinical research has suggested that the interplay between microglia and the GM is crucial for synaptic reorganization during postnatal CNS development [181,182]. In turn, Bruckner et al. [183] noted that compromised social behavior in animal models was linked to a diminishment in neurite complexity and less precise innervation of forebrain neurons. This phenotype was associated with changes in the number of microglial cells and the expression of complement components, specifically C1q. In addition, microglia from GF mice or from those that were treated with antibiotics displayed a non-mature transcriptomic profile, which led to impaired dendritic spine remodeling and impairments in learning [184]. Thus, microglia serve as an important connection between the GM and brain plasticity. Nevertheless, factors such as diet can significantly alter the GM composition [185], subsequently affecting neuronal network remodeling processes by influencing microglial function [186].

In summary, the GM appears to play a pivotal role in healthy brain development, functioning as a major environmental modulator from the prenatal period through early postnatal life by influencing neurogenesis, myelination, BBB integrity, and microglial maturation via the GBA. Key features of GBA development are concisely enumerated as follows. First, critical timing: initial microbial colonization occurs in parallel with rapid brain growth, and early dysbiosis can result in lasting structural and behavioral changes. Second, communication mechanisms: the gut influences the brain through microbial metabolites and neurotransmitters, modulates microglial activity via immune signaling essential for synaptic pruning, and communicates directly with the CNS through neural pathways such as the vagus nerve. Third, developmental impact: evidence from germ-free mice demonstrates that the absence of gut microorganisms leads to behavioral alterations and changes in brain chemistry, including increased motor activity and modified anxiety-like behaviors. Fourth, developmental programming: maternal GM composition substantially shapes offspring brain organization and cognitive development. Building on the aforementioned, it can be asserted that the GM represents an active driver of neurodevelopmental trajectories. Nevertheless, despite compelling experimental evidence, the precise mechanisms and temporal intervals through which the GM exerts its influence remain incompletely understood, particularly in the context of human biological processes. This underscores a need for integrative and longitudinal human studies aimed at elucidating how early-life microbial dynamics interact with physiological and environmental factors to shape developmental outcomes. Ultimately, acknowledging the GM as a fundamental modulator of neurodevelopment opens the possibility for innovative interventions in early life.

## 6. The Gut Microbiome in Immune System Development

The development of the human immune system does not consist of a spontaneous or isolated event. Instead, it is a highly regulated, dynamic, and stepwise process. This process starts in the womb and continues through subsequent life stages. A key component of this development is the microbiome, particularly within the GI [187]. The interaction between the microbiome and immune system is not passive. In fact, it constitutes a mutually reinforcing and ongoing process that is pivotal for establishing immune balance. This equilibrium allows the immune system to differentiate between self and non-self, respond effectively to pathogens, and maintain tolerance toward both self-antigens and commensal microorganisms [188]. In the early stages of life, this symbiotic relationship influences the structure and activity of both primary and secondary lymphoid organs, affects the distinction and stimulation of various immune cell populations, and sets the stage for a resilient, adaptable, and enduring immune response [189]. During this pivotal period, signals from the microbiome may direct aspects of the immune system’s maturation, promoting a balanced and efficient response [190].

### 6.1. Early-Life Gut Microbiome Maturation and Dysbiosis

The GM undergoes rapid transformation during the first three years of life, progressively developing a composition that increasingly resembles that of an adult, with this maturation process generally completed within the first year [191]. In this context, dysbiosis of the GM has been shown to contribute to immune system dysfunction, especially in infants and young children. Preterm infants typically exhibit delayed and irregular GM maturation compared to term infants. In full-term infants, factors such as C-section delivery, early antibiotic exposure, and contact with environmental microorganisms are the primary disruptors of early-life GM development [192]. Research has demonstrated that infants delivered via C-section often exhibit altered microbiomes, including a reduced population of Bacteroidota species [193]. Early antibiotic exposure has been associated with reduced microbial diversity, which may compromise the immune system’s ability to appropriately regulate inflammatory responses [10,194,195]. Early exposure to environmental microorganisms plays a pivotal role in shaping the infant GM [196]. For instance, infants raised in environments with greater microbial diversity, such as on farms or in households with pets, tend to present greater immune system regulation and a lower incidence of conditions such as asthma and allergies. In contrast, being exposed to urban living conditions, processed foods, pollutants, and antibiotics can compromise the epithelial barrier, leading to dysbiosis and increased vulnerability to allergy and autoimmune conditions [193,197,198]. A delayed establishment of a *Bifidobacterium* peak around three months of age has been noted in infants that were exposed to GM disruptors, including preterm birth, C-section delivery, and early antibiotic administration [199]. Although the exact function of this *Bifidobacterium* peak remains to be fully clarified, current evidence suggests that *Bifidobacterium*-derived metabolites may play a role in the development of immune and mucosal barrier functions. For instance, the tryptophan metabolite indole-lactate, produced by *Bifidobacterium* species such as *B. longum*, *B. bifidum*, and *B. breve* during the breastfeeding period, has been shown to activate the AHR and hydroxycarboxylic acid receptor 3, thereby supporting proper mucosal immune development [200,201]. Metabolites derived from *B. longum* subsp. *infantis*, including indole-lactate, have also been reported to influence T cell polarization, providing a molecular link to immune regulation [202]. Disruption of these processes can lead to immune system dysfunction, which has been associated with a range of physiological complications, including metabolic, allergic, and autoimmune disorders. Such complications may manifest as type 1 diabetes mellitus [203], allergies [204], ASD [205], IBD [206], and growth retardation [207]. Table 2 summarizes several pediatric diseases linked to GM dysbiosis.

### 6.2. Impact of Gut Microbial Colonization on Neonatal Immune Development

**Figure 2:**
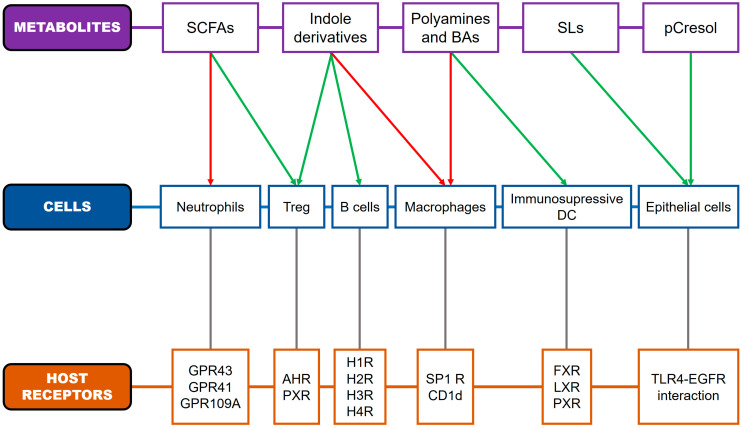
Influence of microbial metabolites on immune cell responses through various host receptors and targets. SCFAs: short-chain fatty acids. BAs: bile acids. SLs: sphingolipids. Treg: regulatory T cell. DC: dendritic cell. GPR: G protein coupled receptor. PXR: pregnane X receptor. HRs: histamine H receptors. SP1: sphingosine-1-phosphate. CD1d: cluster of differentiation 1. FXR: farnesoid X receptor. LXR: liver X receptor. TLR4: toll-like receptor 4. EGFR: epidermal growth factor receptor. Green arrows: activation. Red arrows: inhibition.

The GM establishes complex relationships with both microbial communities and the human host, including symbiotic and sometimes parasitic interactions, which significantly impact the host’s immune system, particularly during early development [216]. In early life, the mucosal immune system detects bacterial presence via pattern recognition receptors (PRRs), such as toll-like receptors (TLRs), which recognize bacterial components, including LPS and flagellin [217]. In humans, TLR10 is primarily expressed in epithelial cells of the small intestine, whereas TLR3 is predominantly localized in colonic epithelial cells, with TLR2 and TLR4 showing lower levels of expression [218]. The concomitant reduction in TLR4 expression and decreased levels of myeloid differentiation-2 (MD2), which constitutes a pivotal co-factor for LPS-mediated signaling, results in intestinal epithelial cells being less responsive to LPS stimulation. Since LPS is a well-known inducer of pro-inflammatory responses, this reduced reactivity is thought to support host-microbial immune homeostasis during the neonatal period, when the intestine is first colonized by microorganisms [218]. Moreover, TLR4-MD2 complexes are confined to the intestinal crypts, reflecting a strategic spatial compartmentalization. This arrangement likely limits immune activation to microorganisms that penetrate the deeper mucosal layers, while maintaining immune tolerance to the majority of commensal organisms residing at the epithelial surface. Therefore, PRRs enable communication between the host and the microbiome, promoting tolerance and diminishing the risk of inappropriate immune responses to harmless microorganisms [219]. Bifidobacteria, for instance, produce exopolysaccharides that influence immune responses in the host, partly through TLR-2 and c-type lectin receptors [220]. Early exposure to LPS triggers a signaling cascade in intestinal epithelial cells, leading to the increased expression of IL-1 receptor-associated kinase 1, which protects cells from bacterial-induced damage [221]. This protective mechanism reduces subsequent immune responses to TLR activation, facilitating microbial colonization and promoting an equilibrated link between the host and its commensal microbiota [216,222]. Substantial distinctions exist in cytokine development between preterm and full-term infants, mainly shaped by their age of gestation and by TLR activation [223]. In preterm infants, TLR activation leads to the production of anti-inflammatory cytokines, such as IL-10, while in full-term infants, cytokines like IL-6 and IL-23 support Th-17 cell development [224]. Besides PRR-mediated recognition of microorganisms, microbial metabolites, including secondary bile acids (BAs), SCFAs, sphingolipids (SLs), tryptophan derivatives, p-cresol, and polyamines, may play a role in establishing immune tolerance and shaping immune networks [225,226]. Figure 2 illustrates how these microbial metabolites influence immune cell responses (modified from [216]).

The early postnatal period represents a critical “window of opportunity” during which the neonatal immune system matures in response to microbial and dietary antigens. This phase is characterized by the establishment of immunological tolerance toward commensal microorganisms, setting the threshold for host-microbiome immune interactions later in life [227]. The early activation and expansion of regulatory T cells (Tregs) are central to preventing excessive immune reactions to the environmental changes that occur after birth. Hayakawa et al. [228] reported that neonates display a marked increase in activated Tregs within the first week of life (7–8 days postpartum) compared to the late neonatal phase (2–4 weeks). Conversely, antenatal exposure to antibiotics significantly reduces the proportion of activated Tregs during the early neonatal period, supporting the notion that early-life microbial encounters drive the development of immunotolerogenic responses [228]. In addition, specific commensal bacteria have been shown to promote the differentiation and proliferation of effector T cells. For instance, *Bacteroides* species, as pioneer colonizers capable of metabolizing human milk oligosaccharides, play a role in regulating T cell differentiation [229]. Monocolonization of GF mice with *Bacteroides fragilis* has been demonstrated to induce immunotolerance via the expansion of FOXP3^+^ T cells in the gut and the production of the immunosuppressive cytokine IL-10 [230]. These immunomodulatory effects are likely mediated, at least in part, by the microbial metabolite indole-3-lactic acid, derived from tryptophan metabolism by *B. fragilis*, which exerts anti-inflammatory actions within the intestinal environment [200].

The early-life expansion of Tregs is tightly controlled by specialized antigen-presenting cells (APCs) expressing RORγt [231]. The involvement of RORγt^+^ innate lymphoid cells (ILCs) in shaping commensal-specific CD4^+^ T cell responses has been examined via major histocompatibility complex class II (MHC-II)-dependent interactions with CD4^+^ T cells. Loss of either RORγt^+^ ILCs or MHC-II expression on these cells disrupts immune regulation toward commensal bacteria, leading to intestinal inflammation in murine models [232]. Notably, MHC-II^+^ ILC3s have been shown to induce apoptosis in activated T cells specific to commensal bacteria. In addition, MHC-II expression on colonic ILC3s is suppressed in children with IBD, indicating a conserved role for these cells across mice and humans [232]. Further research has identified additional RORγt^+^ APC subsets, including AIRE-expressing Janus and Thetis cells and αvβ3 integrin-expressing cells, which populate intestinal lymph nodes during early life. These subsets are both necessary and sufficient to drive the differentiation of RORγt^+^Foxp3^+^ Tregs and to promote tolerance to microbiota-specific T cells [233,234,235].

Both conventional effector T cells and unconventional T cell populations, including invariant natural killer T (iNKT) cells and mucosal-associated invariant T (MAIT) cells, colonize mucosal tissues in response to early-life microbial exposure. The antigenic specificity of these unconventional subsets is limited due to their semi-invariant T cell receptors. In mice, iNKT cells populate the colon within the first 5–6 days after birth, a process that depends on fetal-derived resident macrophages [236]. Studies using GF mice and bacterial monocolonization have demonstrated that early-life GM establishment suppresses colonic iNKT cell expansion, with lasting effects on their function [237,238]. MAIT cells, which are abundant at mucosal sites such as the human gut lamina propria and lungs, recognize metabolites derived from vitamin B2 (riboflavin), which are produced by select intestinal bacteria [239]. Their development in the thymus and subsequent expansion in extrathymic mucosal tissues are dependent on microbiota-derived signals, as evidenced by their deficiency in GF mice and the requirement for riboflavin-producing commensals for proper intrathymic MAIT cell development [240,241]. Although the precise role of MAIT cells in systemic inflammation is not fully defined, emerging evidence highlights their potential contributions to epithelial barrier integrity and wound healing. Moreover, reduced circulating MAIT cell levels have been reported in individuals with various autoimmune conditions [239,242]. Thus, an increasing body of evidence links early-life microbial colonization to the establishment of immune regulation and tolerance toward commensal microorganisms. These findings imply that early microbial exposures may imprint susceptibility to inflammatory responses later in life. Consequently, disruptions to the natural trajectory of postnatal colonization can adversely affect both intestinal and systemic immunity, potentially influencing the development and progression of inflammatory diseases.

In summary, the GM plays a pivotal and irreplaceable role in the development, maturation, and functional programming of the mammalian immune system during early life. Indeed, it serves as an important environmental and epigenetic mediator that transforms an immature neonatal immune system into a functional, tolerant, and protective network. In developmental biology, this interaction is defined by a “window of opportunity”, a critical time period in humans during which microbial colonization and immune education are closely linked. The GM “educates” the immune system through both local mucosal interactions and systemic signaling, ensuring that the host can distinguish between harmless commensals and pathogenic threats. The mechanisms involved in immune system education are as follows. First, innate immune training: PRRs such as Toll-like receptors on intestinal epithelial cells and immune cells (e.g., dendritic cells and macrophages) continuously sense microorganism-associated molecular patterns. Second, adaptive immune maturation: commensal bacteria are essential for the expansion of specialized immune tissues, including Peyer’s patches and isolated lymphoid follicles, which facilitate T and B cell development. Third, metabolite-mediated signaling: SCFAs, including butyrate, acetate, and propionate, act as signaling molecules that support the epithelial barrier and modulate immune cell function. Nevertheless, the full extent to which early-life microbial exposure can be modulated to optimize immune function remains unexplored. From a translational perspective, this raises various possibilities: could customized modulation of the maternal and neonatal GM during the “window of opportunity” be employed to prevent immune-mediated disorders or enhance vaccine responses? Moreover, considering the epigenetic imprinting potential of microbial signals, a future can be proposed in which early microbial interventions shape immediate immune competence and establish long-lasting health trajectories. In this respect, the GM functions as a facilitator of immune maturation but also as a central modulator of developmental adaptability, actively shaping the capacity of the host to respond and function effectively in an environment characterized by a dense, diverse, and interactive community of microorganisms.

## 7. The Gut Microbiome in Neurodevelopmental Disorders

Alterations in the early-life composition of the GM may have enduring effects on neurodevelopment. NDDs constitute a group of conditions that emerge during childhood and interfere with normal brain maturation, consequently affecting cognitive, emotional, and motor capacities [72,243]. The DSM-5 [244] identifies a spectrum of primary NDDs, including ASD, ADHD, motor-related conditions such as tic disorders (TDs) and Tourette syndrome (TS), cerebral palsy, and fetal alcohol spectrum disorders (FASDs). Although the exact underlying mechanisms driving the diverse spectrum of NDDs remain incompletely elucidated, the GBA represents a potential pathway involving endocrine, neural, and immune-mediated domains [245]. Since the interplay between the GM and the brain is established during early childhood and remains susceptible to modulation by factors such as diet, medications, and stress, research underscores the pivotal role that the GM might play in the onset and exacerbation of NDDs [245].

### 7.1. Autism Spectrum Disorder

ASD is defined by impairments in social communication and the presence of restricted and/or repetitive behaviors. Individuals with ASD often exhibit additional behavioral challenges, including aggression, self-injurious behaviors, and heightened irritability [246]. The precise causes of ASD remain unclear, although multiple contributing factors have been proposed, encompassing genetic predispositions, immune dysregulation, environmental influences, and alterations in the GM [247,248,249]. Indeed, evidence has documented changes in the GM of children with ASD [250,251]. These microbial alterations have been associated with neuroinflammatory responses, shifts in neurotransmitter levels, increased oxidative stress, and disruptions in intracellular pH balance.

As noted, research has reported that children with ASD display distinct alterations in their GM. However, the specific taxonomic profiles vary depending on the sequencing methodology employed [252]. For instance, metagenomic analyses have identified mucosal dysbiosis in ASD, characterized by a reduced abundance of Bacteroidota members and an increased Bacillota-to-Bacteroidota ratio. Fecal pyrosequencing has revealed higher levels of *Desulfovibrio* species and *Bacteroides vulgatus* in autistic children compared to neurotypical controls [253]. Additional pyrosequencing research has shown altered microbial diversity in ASD, with increased representation of the families *Sutterellaceae* and *Enterobacteriaceae*, as well as genera such as *Caloramator*, *Akkermansia*, *Sarcina*, *Alistipes*, and *Clostridium*, along with decreased levels of *Coprococcus*, *Prevotella*, and unclassified *Veillonellaceae* [254]. Analyses using 16S rRNA sequencing of the GM from individuals with late-onset autism demonstrated elevated incidences of *Clostridium* and *Ruminococcus* species, whereas real-time PCR highlighted significant abundances of *Clostridium* clusters I and XI and *Clostridium bolteae* [255]. Culture-independent fluorescence in situ hybridization (FISH) research has also reported higher levels of *Clostridium hystolyticum* in children with ASD relative to healthy controls [256]. More recently, Wu et al. [257] applied machine learning techniques to multi-analytic datasets, identifying distinctive microbial signatures in ASD patients. Their findings suggested that the genera *Ruminococcus*, *Prevotella*, *Catenibacterium*, *Roseburia*, and *Megasphaera* could serve as potential microbial biomarkers for ASD. Table 3 summarizes recent studies (2019–2025) investigating GM composition in individuals with ASD.

Bundgaard-Nielsen et al. [258] reported that GM composition in children and adolescents with ASD differs notably from that of typically developing controls. Specifically, individuals with ASD exhibited a reduced relative abundance of *Sutterella*, *Coprobacter*, *Paraprevotella*, and *Bilophila*, along with an elevated presence of *Eggerthella*, *Hungatella*, *Dielma*, and *Ruminococcus gnavus*. Chang et al. [259] conducted a comparative analysis of the GM among individuals with ASD, their unaffected siblings, and healthy controls, also examining correlations between microbial composition and autistic behaviors, emotional/behavioral problems, and GI symptoms. The unaffected siblings group displayed higher alpha-diversity (i.e., bacterial richness and evenness), whereas participants with ASD exhibited distinct beta-diversity (i.e., community composition) patterns in comparison to the control group. Healthy controls were enriched in several microbial taxa, including *Blautia*, *Eubacterium hallii*, *Anaerostipes*, *Erysipelotrichaceae UCG 003*, *Parasutterella*, and *Ruminococcaceae UCG 013*, compared with both ASD and unaffected siblings. In contrast, *Prevotellaceae* at the family level and the genus *Agathobacter* were more prominent in unaffected siblings compared with ASD and control subjects. Notably, variations in microbial composition were associated with autistic and GI symptoms, with higher levels of *Anaerostipes* correlating with reduced social deficits and fewer internalizing behavioral problems. In turn, Chen et al. [260] investigated the GM in an East Asian cohort to determine whether microbial composition is associated with clinical manifestations, emotional and behavioral difficulties, and GI symptoms in individuals with ASD. Compared to healthy controls, participants with ASD exhibited more pronounced GI symptoms. No significant differences were observed between groups in alpha-diversity measures of species richness. Taxonomic analysis revealed that ASD was associated with higher proportional abundances of *Fusobacterium*, *Ruminococcus torques*, and *Bacteroides plebeius*, along with lower proportional abundances of *Ruminococcaceae UCG 013*, *Erysipelotrichaceae UCG 003*, *Parasutterella*, *Clostridium* sensu stricto 1, *Turicibacter*, *Clostridium spiroforme*, and *Intestinimonas butyriciproducens*. Importantly, these microbial alterations were significantly correlated with the severity of autistic traits, including thought problems, delinquent behaviors, self-regulation difficulties, and somatic complaints. Furthermore, Chen et al. [261] examined the relationships between GM composition and both social impairment severity and cognitive performance in children with ASD. Participants were further stratified into two subgroups: those with ASD co-occurring with other developmental disorders and those with ASD alone. Alpha-diversity measures were significantly lower in both ASD subgroups compared with children exhibiting developmental delay or intellectual disability and typically developing controls. At the genus level, children with ASD displayed a reduction in *Prevotella*, while *Bacteroides* and *Faecalibacterium* were elevated compared to children with developmental delay or intellectual disability and healthy controls. Notably, distinct shifts in bacterial composition were linked to the severity of social deficits and performance on intelligence assessments in ASD and its subgroups compared with children with developmental delay or intellectual disability and typically developing controls, suggesting that GM composition may hold potential as a biomarker for ASD-related behavioral and cognitive features.

Recent research has highlighted chronic constipation as one of the most prevalent GI manifestations associated with core symptoms of ASD. To explore the potential interplay between ASD and GI disturbances, Dan et al. [262] conducted a metagenomic analysis comparing children with ASD and chronic constipation with age-matched typically developing individuals. The study revealed that alpha-diversity within the ASD group remained relatively stable across different ages, whereas typically developing children exhibited an age-related increase in alpha-diversity. The constipated ASD cohort displayed reduced microbial diversity and a marked depletion of species including *Prevotella 9*, *Megamonas*, *Ruminococcus 2*, *Sutterella*, *Prevotella*, and *Bacteroides*, along with disruptions in associated metabolic pathways that may contribute to the pathophysiology of chronic constipation in ASD. In addition, differential metabolite analysis indicated alterations in metabolic networks linked to key neurotransmitters, including 5-HT, dopamine, histidine, and GABA, suggesting a potential mechanistic connection between GM dysregulation and neurological signaling in affected individuals.

Ding et al. [263] investigated the associations between clinical manifestations of autistic traits and GI symptoms. The study revealed that individuals with ASD exhibit GM dysbiosis, which may contribute to both the onset of autistic symptoms and GI disturbances. This microbial imbalance was characterized by elevated levels of *Faecalibacterium*, *Prevotella*, *Subdoligranulum*, and *Ruminococcus*, along with a reduced abundance of *Bifidobacterium*. Notably, no significant differences in alpha-diversity were observed between participants with ASD and healthy controls, suggesting that compositional shifts rather than overall microbial richness may underpin the observed symptomatology. In a case–control study, Huang et al. [264] analyzed the GM of individuals with ASD and compared it with age- and sex-matched healthy controls as well as first-degree relatives, using statistical models adjusted for potential confounders such as age. The study revealed a notable enrichment of *Enterobacteriaceae* (e.g., *Escherichia*/*Shigella*) and *Phyllobacterium* in the ASD cohort. Multivariable omnibus analyses identified clinical variables (e.g., behavioral assessments, dietary patterns, GI symptoms) that co-varied with overall GM composition in ASD participants. Moreover, Liu et al. [265] reported that children with ASD exhibited lower fecal concentrations of acetic acid and butyrate, coupled with elevated valeric acid levels. They also observed a reduction in butyrate-producing taxa such as *Ruminococcaceae*, *Eubacterium*, *Lachnospiraceae*, and *Erysipelotrichaceae*, along with an increase in valeric acid-associated bacteria (i.e., Acidobacteria). Among constipated autistic children, GI symptoms correlated with increased abundances of *Fusobacterium*, *Barnesiella*, *Coprobacter*, and other valeric acid-linked taxa (i.e., *Actinomycetaceae*), along with a reduction in butyrate-producing organisms. Ma et al. [266], in turn, found that children with ASD exhibited reduced GM diversity and richness compared with neurotypical peers. After controlling for confounding variables, no significant differences were detected at the phylum level. However, decreased abundances of taxa including *Acidaminococcaceae*, *Lachnoclostridium*, *Tyzzerella* subgroup 4, *Flavonifractor*, and *Clostridium clostridioforme* were observed in ASD participants. Additionally, Niu et al. [267] reported that children with ASD displayed significant differences in microbial evenness and the relative abundance of bacterial phyla and genera compared to healthy controls. At the phylum level, Bacteroidota were reduced in ASD. At the genus level, *Bacteroides*, *Bifidobacterium*, *Ruminococcus*, *Roseburia*, and *Blautia* were significantly less abundant in ASD participants.

Beta-diversity analyses further revealed distinct microbial community structures between ASD and neurotypical children [268]. Controls were dominated by Bacteroidota, whereas ASD participants showed predominance of Bacillota, Actinobacteriota, and Pseudomonadota. Genera such as *Blautia* and *Bifidobacterium* were enriched in controls, whereas *Clostridium* sensu stricto 1, *Ruminococcus torques*, *Lachnospiraceae UCG004*, and *Bifidobacterium breve* were more prevalent in the ASD cohort. Wan et al. [269] determined that ASD status and chronological age exerted the strongest influence on children’s GM, whereas dietary factors showed no significant correlation. Compared with controls, children with ASD exhibited increased bacterial richness and altered microbial community composition. Specific bacterial species were identified as discriminators between ASD and control microbiota (Table 3). Furthermore, multiple neurotransmitter biosynthesis pathways were depleted in ASD-associated GMs.

Wang et al. [270] observed significant differences in both alpha- and beta-diversity between ASD and control groups. Dominant taxa in neurotypical children included *Flavonifractor* and *Bradyrhizobium*, whereas ASD participants showed higher abundances of *Streptococcus*, *Ruminococcus*, and *Ruminiclostridium*. Metabolic pathways associated with alpha-linolenic acid were reduced in the GM of ASD subjects, whereas pathways related to uncharacterized conserved proteins, aminoglycoside phosphotransferase, and inorganic pyrophosphatase were elevated compared to controls. A pilot study aiming to explore trace element and GM profiles in Chinese children with ASD identified significantly higher concentrations of lead, arsenic, copper, zinc, mercury, calcium, and magnesium in the ASD group [271]. Linear discriminant analysis revealed elevated abundances of nine bacterial genera, including *Bacteroides*, *Parabacteroides*, *Sutterella*, *Lachnospira*, *Bacillus*, *Bilophila*, *Lactococcus*, *Lachnobacterium*, and *Oscillospira*. Redundancy analysis highlighted significant associations between arsenic and mercury with *Parabacteroides* and *Oscillospira*.

Zou et al. [272] examined GM alterations in Chinese children with ASD. At the phylum level, Bacillota, Pseudomonadota, and Verrucomicrobiota were decreased, whereas the Bacteroidota/Bacillota ratio was elevated due to increased Bacteroidota. At the genus level, *Bacteroides*, *Prevotella*, *Lachnospiraceae incertae sedis*, and *Megamonas* were enriched, while *Clostridium XIVa*, *Eisenbergiella*, *Clostridium IV*, *Flavonifractor*, *Escherichia*/*Shigella*, *Haemophilus*, *Akkermansia*, and *Dialister* were reduced. Notably, species involved in branched-chain amino acid synthesis (e.g., *Bacteroides vulgatus*, *Prevotella copri*) were increased, whereas *Bacteroides fragilis* and *Akkermansia muciniphila* decreased, suggesting that microbial metabolic dysregulation may contribute to ASD pathophysiology. Moreover, Zuffa et al. [273] conducted a prospective longitudinal study tracking fecal microbiota and metabolome development in infants with or without a family history of ASD during the first 36 months of life. At five months, infants at elevated ASD likelihood exhibited lower *Bifidobacterium* and higher *Clostridium* and *Klebsiella* levels compared to low-likelihood infants. Untargeted metabolomics showed that low-likelihood infants excreted higher levels of fecal GABA, which declined over time. Integrated microbiome-metabolome analyses revealed positive correlations between GABA and *Bifidobacterium*, and negative associations with *Clostridium*.

It has been noted that gut microbial communities produce a variety of metabolites and neurotransmitters capable of crossing both the intestinal barrier and the BBB, thereby influencing CNS function. These microbial products may modulate mitochondrial activity and affect the epigenetic regulation of genes associated with ASD [274]. The impact of SCFAs on ASD, however, remains a topic on which consensus has not yet been reached. In preclinical studies, propionate has been shown to induce hippocampal gene expression changes, histological alterations, and ASD-like behavioral abnormalities, including repetitive behaviors and deficits in social interaction [275]. In contrast, butyrate has demonstrated beneficial effects in ASD-like murine models, ameliorating social and repetitive behavioral phenotypes [276]. Notably, butyrate has been reported to restore BBB integrity and counteract propionate-induced abnormalities [277]. On the other hand, human studies have yielded mixed findings. De Angelis et al. [254] observed elevated fecal levels of propionate and acetate, along with reduced butyrate concentrations in children with ASD. Conversely, Liu et al. [265] reported decreased acetate and butyrate levels but increased fecal valerate in ASD subjects, along with a reduced abundance of butyrate-producing taxa, such as *Eubacterium*, *Ruminococcaceae*, *Erysipelotrichaceae*, and *Lachnospiraceae*, and increased valerate-associated bacteria, including members of Acidomycetota. In turn, Wang et al. [278] found no significant correlation between SCFA concentrations and ASD risk.

Evidence suggests a link between microbial-derived neurotransmitters and the manifestation of ASD-related symptoms. Garcia-Gutierrez et al. [279] reported that core ASD symptoms, including anxiety, cognitive impairments, and conduct disturbances, may be associated with the relative abundance of *Bifidobacterium* and *Bacteroides*, which play essential roles in GABA synthesis [117]. Furthermore, the presence of neurotoxin-producing species, such as *Clostridioides difficile* and *C. histolyticum*, has been correlated with ASD phenotypes, as described by Sivamaruthi et al. [252]. These microbial toxins are capable of interfering with 5-HT signaling, potentially contributing to reduced social interaction, atypical language acquisition, altered pain perception, and the emergence of repetitive or self-injurious behaviors [251,280]. In addition, elevated CNS glutamate levels have been documented in children with ASD, implicating excitatory neurotransmission in the pathogenesis of several NDDs [281]. Altered concentrations of p-cresol and p-cresyl sulfate, which inhibit dopamine-β-hydroxylase activity, have also been observed in ASD patients [282]. Such disruptions in neurotransmitter metabolism may underlie the behavioral and cognitive deficits characteristic of ASD [283].

All these findings indicate that early-life alterations in the GM may precede behavioral manifestations in ASD, suggesting a role for microbial composition in shaping subsequent symptom variability. In this context, the GM also appears to influence fecal SCFA profiles and constipation, highlighting its functional impact on GI health. Considering that the developmental trajectory of the GM in ASD diverges from that observed in typical development, these insights underscore the potential contribution of both the GM and its metabolites in ASD pathogenesis. Nevertheless, further research is needed to determine the precise roles of microbial alterations, GI permeability, and immune dysregulation in the behavioral, immunological, and GI features of ASD. Ultimately, this emerging evidence invites a broader perspective on autism, positioning it as a condition potentially shaped by early-life gut microbial dynamics.

### 7.2. Attention-Deficit/Hyperactivity Disorder

ADHD is defined by patterns of inattention, hyperactivity, and impulsivity that are inappropriate for a child’s developmental stage and result in functional impairment [284]. The trajectory of ADHD can vary, with childhood risk factors influencing whether symptoms remit or persist into adulthood [285]. Although the underlying causes of ADHD remain incompletely understood, emerging evidence suggests that early-life GM composition and dietary influences on microbial communities may contribute to the development and expression of ADHD symptoms [286,287,288].

A systematic review into the GM of individuals with ADHD revealed heterogeneous findings [289]. Overall, research reports reductions in both bacterial richness and evenness (alpha-diversity), as well as alterations in community composition (beta-diversity), in affected individuals [290]. Another systematic review identified an increased relative abundance of *Odoribacter* and *Eggerthella*, along with a decreased presence of *Faecalibacterium* in ADHD patients [291]. Table 4 summarizes recent studies (2019–2025) investigating GM composition in individuals with ADHD.

Bundgaard-Nielsen et al. [258] reported that children and adolescents diagnosed with ADHD exhibit GM profiles distinct from non-related control subjects. Specifically, reductions were observed in the genera *Coprobacter*, *Bilophila*, *Howardella*, and *Colidextribacter*, while *Streptococcus*, *Lactobacillus*, *Hungatella*, *Eggerthella*, and *Ruminococcus gnavus* were increased. A cross-sectional analysis by Cassidy-Bushrow et al. [286] also suggested that GM composition differs between individuals with and without ADHD. Their investigation indicated that microbial patterns at one and six months of age were associated with ADHD diagnosis by age ten. Lee et al. [292] explored potential associations between GM and emotional-behavioral comorbidities commonly observed in ADHD. Adjusted analyses revealed that *Agathobacter* was significantly linked to withdrawal and depression symptoms, while *Ruminococcus gnavus* correlated with rule-breaking behaviors. To further clarify GM-ADHD relationships, Richarte et al. [293] compared the microbiota of 100 medication-naïve adults with ADHD to 100 sex-matched healthy controls, reporting notable differences in the relative abundance of multiple microbial taxa in the ADHD cohort. Moreover, Steckler et al. [294] evaluated GM composition and SCFA profiles in children aged 6–18 years with ADHD compared to healthy peers. They identified distinct metabolite signatures in ADHD, emphasizing the potential utility of GM and SCFA profiling for both diagnosis and therapeutic intervention.

Szopinska-Tokov et al. [295] investigated the link between GM composition and core ADHD symptoms of inattention and hyperactivity/impulsivity. Beta-diversity analyses revealed significant differences in bacterial communities between ADHD participants and controls, particularly associated with inattention. Among ten genera showing nominal differences, variation in *Ruminococcaceae UCG 004* correlated with inattention scores, reinforcing the potential role of GM in ADHD pathophysiology. In a case–control study, Wan et al. [296] examined GM composition in children with ADHD and explored its involvement in disease mechanisms. No significant differences in alpha-diversity were observed, although they found that ADHD participants had decreased *Faecalibacterium prausnitzii*, *Ruminococcus gnavus*, *Lachnospiraceae*, and *Veillonellaceae*, along with increased *Bacteroides caccae*, *Odoribacter splanchnicus*, *Paraprevotella xylaniphila*, *Veillonella parvula*, and *Enterococcus*. In turn, metabolic pathway analyses indicated differences in neurotransmitter-related pathways, including 5-HT and dopamine. Wang et al. [288] applied linear discriminant analysis effect size to identify taxa differentially enriched between ADHD patients and controls. They found decreased *Bacteroides coprocola* and increased *B. uniformis*, *B. ovatus*, and *Sutterella stercoricanis* in ADHD subjects. Notably, *S. stercoricanis* abundance correlated with dietary intake (e.g., dairy, nuts, seeds, legumes) as well as ferritin and magnesium levels, and both *S. stercoricanis* and *B. ovatus* were positively associated with ADHD symptom severity. Another study by Wang et al. [297] investigated GM imbalance and inflammatory markers in ADHD. While alpha- and beta-diversity did not differ significantly from controls, five taxa (i.e., *Agathobacter*, *Anaerostipes*, *Lachnospiraceae UCG-010*, *Roseburia*, and *Ruminococcaceae*) were differentially abundant. Furthermore, TNF-α levels were inversely correlated with GM diversity and ADHD symptom severity. On the other hand, Wang et al. [298] reported significant ADHD-associated differences in both alpha- and beta-diversity metrics. Key species contributing to these differences included *Lachnospira* spp., *Phascolarctobacterium faecium*, *Anaerostipes hadrus*, *Odoribacter splanchnicus*, *Alistipes onderdonkii*, *Alistipes shahii*, *Megamonas funiformis*, and *Bacteroides caccae*.

Research has highlighted links between specific microbial taxa, ADHD symptomatology, and disruptions in metabolic pathways. Aarts et al. [299] reported a relationship between *Bifidobacterium* abundance and the presence of enzymes involved in the phenylalanine metabolic pathway in individuals with ADHD. Swann et al. [300] identified metabolic alterations in fecal samples from ADHD patients, which have been previously associated with behavioral manifestations characteristic of the disorder. Research has also observed negative correlations between hyperactivity and *Faecalibacterium* abundance [290]. In addition, *Bacteroides* levels have been associated with hyperactive and impulsive behaviors in children with ADHD [288,290].

Alterations in neurotransmitter systems, including GABA, dopamine, norepinephrine, and 5-HT, have been implicated in dysregulation of brain networks associated with ADHD. These disruptions are linked to impaired reward processing, deficits in attention and working memory, and inadequate behavioral control manifesting as hyperactivity and impulsivity [301]. As previously stated, the GM interacts with the CNS by producing neurotransmitters, their precursors, or other metabolites. Elevated dopamine transporter density observed in ADHD patients can modify synaptic dopamine levels, thereby influencing cognitive function [302]. Notably, 5-HT is involved in regulating mood and inhibitory control, and interventions that enhance serotonergic signaling have been shown to alleviate hyperactivity and impulsive behaviors in ADHD [303]. Likewise, GABA serves as a key regulator of neuronal excitability, with reduced GABA concentrations being associated with increased impulsivity in individuals with ADHD [304].

Research indicates that individuals with ADHD display lower concentrations of SCFAs in both plasma and fecal samples compared to neurotypical controls. In this context, propionic acid levels have been inversely linked to the severity of ADHD symptoms, suggesting its potential utility as a biomarker [305,306]. Butyrate, in turn, exhibits anti-inflammatory effects by suppressing the production of pro-inflammatory cytokines, including IL-6 and IL-12, while promoting the release of the anti-inflammatory cytokine IL-10 [307]. Furthermore, psychostimulant medications commonly used to manage ADHD have been shown to alter GM composition and SCFA concentrations, which may influence the GBA and potentially modulate therapeutic outcomes [305].

Overall, these findings indicate that children and adults with ADHD exhibit distinct GM profiles compared to healthy controls, with alterations observed in both microbial composition and associated metabolites, including SCFAs. Specific genera, such as *Ruminococcus gnavus*, *Agathobacter*, *Faecalibacterium*, and *Bacteroides* species, have been implicated in ADHD, and changes in these taxa have been linked to core symptoms. In addition, diet constitutes an important modulator of the GM, interacting with microbial composition to potentially influence ADHD susceptibility, as highlighted by associations between certain taxa, dietary patterns, and symptom severity. Altered neurotransmitter metabolic pathways could also serve as potential contributing factors to the symptom profile observed in ADHD. Despite these observations, the underlying mechanisms remain incompletely understood, and further studies are required to clarify how GM alterations, GI permeability, and immune dysregulation collectively contribute to the features observed in pediatric ADHD populations.

### 7.3. Tic Disorders and Tourette Syndrome

TDs represent a subgroup of NDDs that typically arise during childhood and are characterized by the abrupt onset of repetitive, nonrhythmic movements, which may persist into adulthood [308]. It can be argued that TS constitutes the most severe form of TDs. Specifically, TS is recognized as a chronic NDD that commonly emerges in childhood and is defined by the presence of both motor and phonic tics, often imposing a considerable burden on affected individuals’ quality of life [309]. While many patients experience spontaneous remission before reaching adulthood, approximately one-third continue to exhibit symptoms throughout life. Those with severe TS may present with uncontrollable behaviors and vocalizations, including coprolalia, significantly affecting social interactions and overall well-being [310]. Although the precise pathophysiological mechanisms underlying TS are yet to be fully understood, multiple studies have suggested the involvement of genetic, neurobiochemical, immunological, microbial, and environmental factors [311,312,313,314].

Recent literature reviews have investigated the potential association between GM alterations and the severity of TS symptoms [315,316]. However, the studies reviewed did not reach a consensus regarding changes in alpha- or beta-diversity. One study reported a reduction in both the family *Prevotellaceae* and the genus *Prevotella* among individuals with TS, accompanied by an increased abundance of *Ruminococcus*. At the species level, decreases in *Clostridium bartlettii*, *Prevotella copri*, and *Subdoligranulum variabile* were also observed [317]. Xi et al. [318] demonstrated that treatment with dopamine receptor antagonists could reverse GM dysbiosis. In treatment-naïve children, higher levels of *Phocaeicola* (formerly *Bacteroides*) *plebeius* and *Mediterraneibacter faecis* (formerly *Ruminococcus lactaris*) were detected, suggesting that these bacterial taxa and their metabolites may contribute to oxidative stress and inflammatory processes. Furthermore, GABA degradation was significantly increased in children with TS, with *Klebsiella pneumonia*, which constitutes a bacterium implicated in GABA breakdown, positively correlating with symptom severity. Conversely, taxa associated with GABA production, such as *Eubacterium* spp., *Bifidobacterium* spp., and *A. muciniphila*, exhibited a negative correlation with scores on the Yale Global Tic Severity Scale (YGTSS) [316].

### 7.4. Cerebral Palsy and Epilepsy

Cerebral palsy is recognized as the most common childhood motor disability, primarily affecting movement and posture [319]. Clinical manifestations of cerebral palsy are highly variable, but typically involve permanent impairments in motor development that lead to functional limitations [320]. Approximately 40% of individuals with cerebral palsy also present with comorbid neurological disorders, such as epilepsy, with the prevalence of epilepsy in this population reported to be five times greater than in healthy children [321]. Zelnik et al. [322] identified key risk factors for cerebral palsy with epilepsy (CPE), including neonatal seizures, low birth weight, intracranial hemorrhage, lesions in gray and white matter, and structural brain malformations.

Comparative analyses of the GM have shown that children with CPE present with greater microbial diversity and distinct bacterial profiles relative to healthy controls [323]. However, it remains difficult to determine whether these microbial differences are directly attributable to CPE itself or are influenced by lifestyle and dietary factors associated with these neurological conditions [324]. In this respect, both studies consistently reported alterations in the GM of CPE patients [323,324]. At the phylum level, an increased relative abundance of Actinomycetota and a decreased abundance of Bacteroidota were observed. At the genus level, beneficial bacteria such as *Bifidobacterium*, along with opportunistic pathogens, including *Parabacteroides*, *Enterococcus*, and *Streptococcus*, were elevated, while butyrate-producing genera such as *Bacteroides*, *Ruminococcus*, *Faecalibacterium*, and *Roseburia* were reduced. These shifts in microbial composition have been associated with GI consequences, such as chronic intestinal inflammation and functional constipation [325], as well as diminished biosynthesis of secondary microbial metabolites [326]. Functional pathway analyses revealed a reduction in GM pathways involved in the degradation of serine, quinolinic acid, glutamate, and glycerol, along with an increase in pathways related to the dissimilatory reduction of sulfate and nitrate and ethanol production. Notably, both glutamate and serine act as agonists of N-methyl-D-aspartate (NMDA) receptors, and the abnormal expression or activity of these receptors has been proposed as a potential contributor to the pathophysiology of seizures and epilepsy [327,328]. Table 5 summarizes recent studies (2019–2025) investigating GM composition in individuals with TDs, TS, and CPE.

In the context of TD, Wang et al. [329] highlighted that examining shifts in GM structure and diversity could offer valuable clinical insights for both diagnosis and management of children with this particular condition. Complementing this, Xi et al. [318] conducted an exploratory investigation demonstrating that individuals with TD present a distinct GM composition. Specific bacteria, such as *Mediterraneibacter faecis* (previously *Ruminococcus lactaris*), which are enriched in individuals with TD, exhibit pro-inflammatory properties that might contribute to the neuroinflammatory processes observed in some cases. In addition, alterations in microbial metabolic activities related to neurotransmitter pathways, including GABA degradation, may further exacerbate neurotransmitter system dysfunction in TD. Bao et al. [330] characterized the GM in children with TS and evaluated the potential impact of a combined physiotherapeutic intervention, consisting of 60 min of cranial electrotherapy stimulation followed by 30 min of biofeedback training, on microbial composition. Their findings revealed a recognizable microbial signature in children with TS, with certain pro-inflammatory taxa potentially driving neuroinflammatory responses. In children with CPE, Huang et al. [323] illustrated the GM landscape, detailing bacterial interactions and functional capacities within these microbial communities, as well as underscoring the potential involvement of the GM in CPE pathogenesis. Moreover, Peng et al. [326] reported evidence of GM dysbiosis in children with CPE, identifying alterations in microbial species, functional pathways, and metabolite profiles. Integrated metagenomic and metabolomic analyses highlighted potential neuroprotective roles for *Bacteroides fragilis* and *Dialister invisus*, while also implicating gut–brain axis mechanisms in the dysregulation of kynurenine, 5-HT, and dopamine pathways, which interact with neuroimmune and neuroendocrine networks.

Overall, current evidence suggests that GM composition and metabolism, particularly involving pro-inflammatory taxa and neurotransmitter-related pathways, may influence neurodevelopmental disorders such as TD, TS, and CPE. Nevertheless, most studies remain exploratory, while the heterogeneity of patient populations, profiling methods, and functional readouts further complicates interpretation. Consequently, rigorous longitudinal and mechanistic studies with human populations are needed to clarify these relationships and translate them into clinical strategies.

### 7.5. Fetal Alcohol Spectrum Disorders

FASDs comprise a range of developmental impairments in children resulting from prenatal exposure to maternal alcohol consumption. The spectrum encompasses four distinct clinical entities: fetal alcohol syndrome, partial fetal alcohol syndrome, alcohol-related neurodevelopmental disorder, and alcohol-related birth defects [331,332]. Diagnosis of FASDs is based on four core criteria: characteristic facial dysmorphology, growth deficits, CNS dysfunction, and confirmed prenatal alcohol exposure. These disorders are associated with structural and developmental abnormalities in the brain, alterations in white matter microstructure, and disruptions in functional connectivity [333]. Consequently, affected individuals often exhibit deficits across multiple domains, including cognition, executive functioning, memory, sensory processing (e.g., vision and hearing), motor skills, behavior, and social adaptation [334].

Alcohol easily crosses the placental barrier, posing significant risks to fetal development. The severity of prenatal alcohol exposure (PAE), which underlies FASDs, depends on multiple factors, including the amount, pattern, timing, and duration of alcohol consumption. Additional determinants include the genetic background of both the mother and fetus, maternal nutrition, concurrent substance use, and epigenetic responses [335]. PAE has been shown to adversely affect neurodevelopment, resulting in a spectrum of structural abnormalities such as microcephaly, hydrocephalus, corpus callosum malformations, prenatal ischemic lesions, small subarachnoid heterotopias, holoprosencephaly, and lissencephaly [336,337]. Preclinical studies further indicate that PAE can induce lasting changes in the GM, often accompanied by increased alpha- and beta-diversity [338,339]. Nevertheless, clinical studies in humans are essential to confirm whether these microbiome alterations are causally linked to PAE-related outcomes.

## 8. The Gut Microbiome in Early-Life Mental Health

The GBA seems to be central to mental health, but evidence is still limited, especially during critical developmental periods characterized by high neural plasticity, when mental health disorders such as anxiety and depression often first emerge [340]. The multidimensional interplay between the CNS and the microbiome further challenges efforts to fully elucidate these interactions. Early childhood represents a phase of rapid GM maturation, potentially constituting a sensitive period during which microbial signals can influence key neurodevelopmental processes [341]. These early microbial influences may have long-lasting effects, shaping neurobiological systems in ways that either heighten vulnerability or confer resilience to later mental health outcomes, particularly during the elevated risk period of middle childhood [342].

Emerging evidence underscores the importance of elucidating the role of the GBA in shaping mental health, particularly regarding neurocognitive and behavioral outcomes during childhood, as reflected in associations with internalizing symptoms [343,344]. Nevertheless, most research investigating the GBA in typical development has concentrated on early childhood and infancy, with a primary focus on neurocognitive measures rather than mental health outcomes [345,346,347]. Current research indicates that individual variability in microbial alpha-diversity is linked to structural and functional differences in brain regions and networks involved in sensorimotor processing, language, and emotional regulation [345,346,347,348]. For instance, higher alpha-diversity in infancy has been associated with increased fronto-parietal connectivity, constituting a network involved in cognitive control, which correlates with elevated negative emotionality in infants [347]. In contrast, alpha-diversity at one year of age has been associated with reduced functional connectivity between the left amygdala and thalamus and between the anterior cingulate cortex and insula, along with enhanced connectivity between the supplementary motor area and inferior parietal lobule [348]. Moreover, greater alpha-diversity at one year has been linked to poorer cognitive performance in domains such as visual reception and expressive language, as well as alterations in brain volume at later stages. Specifically, increased volumes of the left precentral gyrus, right angular gyrus, and left amygdala were observed at age two, highlighting potential early microbiota-driven programming effects on brain development [345]. Despite these findings, the extent to which early-life microbial diversity and composition influence brain activity during middle childhood, which is a period of heightened vulnerability for mental health disorders, remains largely unresolved [349].

Depression is a polygenic psychiatric disorder that continues to impose a substantial societal burden. Antidepressants, particularly selective serotonin reuptake inhibitors (SSRIs), are among the most commonly prescribed medications worldwide. Since the formulation of the monoamine hypothesis of depression, accumulating research has expanded this theory to include additional perspectives, notably the neurotrophic and neurogenesis hypotheses. These expanded theories suggest that reductions in neurotrophic factors such as BDNF or a decline in adult hippocampal neurogenesis contribute to the pathophysiology of depression [350,351]. The role of 5-HT signaling and 5-HT receptors in regulating neurotrophic factors and hippocampal neurogenesis has been a major focus of research [352]. It is widely accepted that neurogenesis in mammals occurs in two distinct regions: the subventricular zone (SVZ) of the lateral ventricle and the subgranular zone (SGZ) of the dentate gyrus (DG) in the hippocampus [353]. Neurons generated in the SVZ migrate along the rostral migratory stream to the olfactory bulb, where they differentiate into interneurons. Conversely, neurons originating in the SGZ migrate into the granular layer of the DG, eventually maturing into granule neurons. Adult neurogenesis involves several stages, including the proliferation and fate specification of neural progenitors, neuronal migration and maturation, and the integration of newly formed neurons into existing synaptic networks. Although the enhancement of hippocampal neurogenesis is pivotal for the antidepressant effect, it alone is insufficient to elicit a full antidepressant response. Moreover, there is limited evidence to support the notion that reduced adult hippocampal neurogenesis directly contributes to the pathophysiology of depression [352].

The role of adult neurogenesis in hippocampal function within the primate brain has been a topic of considerable debate, with recent findings suggesting that this process may play an important role in human cognition [354]. The ventral region of the hippocampus is thought to be particularly involved in mood regulation and emotional behaviors, due to its connections with the hypothalamus, nucleus accumbens, and amygdala. Indeed, research indicates that chronic stress has a more pronounced impact on the ventral hippocampus [355]. The effects of stress are primarily mediated through the HPA axis and subsequent production of glucocorticoids. Elevated levels of corticosterone alone appear to be sufficient to induce behaviors resembling depression and anxiety [356,357]. On the other hand, research has shown that damage to the ventral hippocampus is linked to increased anxiety-like behaviors [358] as well as depression-like symptoms [359]. The dorsal hippocampus, in contrast, is involved in certain cognitive processes, such as memory, learning, and spatial navigation, through its connections with various cortical regions [360]. The hippocampus plays a pivotal role in these cognitive functions due to its ability to undergo both structural and functional synaptic plasticity in response to external stimuli [353].

Maternal stress has been shown to significantly influence the development of the fetal brain, increasing the child’s susceptibility to cognitive impairments. One of the most affected regions is the PFC, which undergoes an extended period of development and is particularly vulnerable to prenatal stress [130]. Suwaluk et al. [361] found that prenatal stress hindered the progress of PFC development by impairing pivotal components of GABAergic processes. These changes are thought to contribute to the emergence of anxiety during adolescence, potentially facilitating the onset and exacerbation of mental health disorders. In addition, GABAergic signaling in the amygdala has been shown to play a key role in regulating emotional processing. Mice with elevated anxiety levels exhibited increased expression of GABA-synthesizing enzymes, such as glutamate decarboxylase, suggesting a compensatory activation of GABA transmission within the amygdala [362]. Disturbances in the early stages of brain development have been linked to the onset of anxiety-like behaviors in adulthood, with associated alterations in genes related to neurotransmission within the amygdala. These changes result in gene expression patterns similar to those observed in psychiatric disorders related to anxiety. Notably, genes involved in synaptic transmission were upregulated, while genes associated with emotion, social behavior, and brain development showed reduced expression [363]. Research has established a positive correlation between anxiety-related behaviors and deficits in inhibitory neurotransmission during development. In this context, enhancing GABAergic activity has the potential to modulate the developmental programming of anxiety-related behaviors [364]. Furthermore, chronic deficits in TGF-β have been linked to increased expression of GABAARα2, disrupting the balance between excitation and inhibition in the basolateral amygdala circuits. This disruption seems to be a significant mechanism underlying the development of anxiety disorders following neonatal inflammation [130].

## 9. Final Remarks

The development of the brain during the fetal stage and the early years of life is particularly susceptible to external influences. As a result, adverse events during this critical period may lead to changes in brain structure and function, potentially increasing the risk of metabolic conditions and NDDs [3,365]. The interplay between the GM and the brain begins to form during early childhood and is shaped by a range of factors, including pharmaceuticals, diet, and stress throughout life [17,185,366]. The mechanisms by which the GM can influence brain-related outcomes are diverse, involving both the nervous and immune system, tryptophan metabolism, and the HPA axis [11]. However, establishing a clear cause-and-effect relationship between GM alterations and disease remains challenging, as it is difficult to determine whether changes in the GM are a causal factor or a consequence of the condition. Therefore, a deeper understanding of how the GM might affect brain-related and developmental outcomes is pivotal for its implications in both clinical and social sciences. Although the mechanisms through which bacteria and their metabolites influence brain function are not fully understood, advances in metabolomics technology offer promise in addressing this gap. In particular, the emergence of high-throughput sequencing technologies could significantly enhance our understanding of the genetic foundations of the GM and its connection to brain-related disorders [367]. Furthermore, the development of new non-invasive sampling techniques will enable the collection of luminal contents from across the intestinal tract, offering an alternative to the limitations of stool sampling [368]. In this context, Maes et al. [369] posited a concept called “precision nomothetic psychiatry”, which aids in exploring causal relationships between recurrence of illness index (ROI), causome/protectome factors, cognitive impairments, and a quantitative measure of the phenome of depressive disorder. This model demonstrates that adverse childhood experiences (ACEs) and the heightened translocation of Gram-negative bacteria are strongly associated with depressive disorders. Moreover, these outcomes are modulated by ROI, diminished antioxidant defenses (e.g., reduced high-density lipoprotein cholesterol), and the stimulation of immune and oxidative stress pathways. Despite the aforementioned, there is still no evidence showing whether ACEs and ROI influence the GM or whether GM dysbiosis could modulate the impact of ACEs on depressive disorders, particularly regarding cognitive impairments and suicide-related behaviors.

### 9.1. Perspectives on Microbiome-Driven Development

The interplay between the human immune system and the microbiome has become one of the most extraordinary findings in 21st-century biology, significantly reshaping the overall understanding within physiology-related fields and also the origins of many health conditions. As previously underscored, research indicates that the GM plays essential roles in immune modulation, epithelial integrity maintenance, and systemic homeostasis. Dysbiosis has been linked to a variety of clinically significant outcomes in infectious, chronic, autoimmune, oncological, and neurological disorders [190,370]. Indeed, recent studies have highlighted the GM as a pivotal modulator of immune cell maturation, cytokine production, and epithelial barrier activity [371]. This has induced the application of therapies using probiotics, prebiotics, synbiotics, and FMT, all of which have demonstrated promising outcomes in reestablishing microbial equilibrium and modulating immune activity [372,373]. In addition, gene-editing technologies, notably CRISPR-Cas, have recently emerged as precise tools for manipulating microbiome composition and function. These tools can target and eliminate antibiotic resistance genes, such as *blaNDM-1* and *mcr-1*, without disturbing the overall microbial ecosystem. Moreover, they can be used to modify microbial metabolic pathways for therapeutic purposes [374,375]. Recent advancements in CRISPR technology have demonstrated its transformative potential for microbiome engineering, including efficient base editing in gut *Bacteroides* species, enabling specific nucleotide changes, multiplex editing of carbohydrate metabolism genes, and expanding the tool’s use across various commensal species [376,377]. Furthermore, microbial metabolites, particularly SCFAs, have been shown to influence the immune system by fostering the differentiation of regulatory T cells, decreasing pro-inflammatory cytokines such as IL-6 and TNF-α, and enhancing the epithelial barrier through proteins such as claudins and ZO-1. Notably, disruptions in these processes have been associated with a range of inflammatory, autoimmune, metabolic, and neurodegenerative diseases [378]. Nevertheless, the routine clinical use of these interventions remains limited, as current evidence for efficacy, safety, and optimal implementation (e.g., dosing, target population) is still under investigation.

In developmental research exploring the link between the GM and brain function, most studies have relied on seed-based resting-state functional connectivity (RSFC) analyses [345,348]. RSFC is advantageous in pediatric populations due to its minimal task demands. However, the use of researcher-selected seeds limits the scope of inference regarding brain function. This approach does not capture connectivity patterns within or between canonical large-scale functional networks, which have been implicated in neurocognitive and psychopathological outcomes in children [379,380]. For instance, alterations in RSFC within the default mode network (DMN), the ventral attention network, and between the DMN and striatal-cingulo-opercular networks have been associated with internalizing symptoms in youth [379]. Across development, a consistent trajectory of increasing within-network integration, along with enhanced between-network segregation, has been documented, supporting the emergence of specialized network functions and efficient information processing. Deviations from this trajectory have been linked to heightened risk for psychopathology in children and adolescents [381,382]. Therefore, investigating how early-life GM influences the maturation of brain network integration and segregation is essential for understanding its role in mental health outcomes. Addressing this question is challenging due to the high-dimensional nature of both GM composition and large-scale network connectivity. Nevertheless, multivariate machine learning techniques offer a promising approach to overcoming these analytical complexities.

The GM also influences neuroimmune functions through the GBA, where metabolites such as tryptophan derivatives and propionate may play key roles in modulating behavior, neuroinflammation, and neuronal plasticity. This has opened new avenues for interventions based on psychobiotics, FMT, and dietary patterns [383,384,385]. Despite these advancements, the clinical implementation of GM-targeted interventions implies substantial challenges, including limited predictive capacity, considerable interindividual variability, an incomplete understanding of host-GM interactions, regulatory and ethical issues, and issues regarding the standardization of therapeutic approaches like FMT [386]. Overcoming these obstacles will require the development of novel technologies for accurate and context-specific GM profiling, integrating artificial intelligence and machine learning to analyze large-scale clinical and multi-omics data [387]. In turn, strong predictive approaches that combine transcriptomic, genomic, metagenomic, and metabolomic data will be essential to optimize therapeutic approaches, enhance efficacy, and minimize potential side effects [388]. Thus, future research should focus on longitudinal pediatric cohorts that assess GM composition, immune development, and neurodevelopmental outcomes across multiple early-life stages. Integrating multi-omics datasets with longitudinal clinical and environmental data will be pivotal to identify causal relationships, temporal dynamics, and specific biomarkers. Although pediatric studies using GM-targeted interventions seem promising, evidence regarding their current clinical application remains limited, with many questions still unresolved, including factors influencing outcomes, duration of effects, safety concerns, and interactions with other treatments [384].

Finally, it is important to note that perturbations of the GM, including those induced by psychoactive substances, might exert effects on neurodevelopment and immune system maturation, further highlighting the role of the GM as a mediator of environmental influences on brain and systemic health. Substance use, even at moderate levels, can induce dysbiosis, compromise gut barrier integrity, and trigger inflammatory cascades, thereby affecting reward circuits, neuroimmune signaling, and behavioral outcomes [389]. These effects may be particularly consequential during early developmental stages, including prenatal life, when the fetal brain and immune system are highly sensitive to environmental perturbations. Consequently, maternal microbiome alterations or substance-induced dysbiosis may have lasting impacts on offspring neurodevelopment, immune function, and susceptibility to NDDs, which emphasizes the critical importance of understanding microbiome-environment interactions during these periods of particular vulnerability.

### 9.2. Current Challenges in Human Microbiome Research

Establishing causality in microbiome research remains a significant challenge, primarily due to three interrelated factors: the inherent complexity of microbial ecosystems, pronounced inter-individual variability, and the difficulty in determining whether microbial alterations are causative or consequential in disease. Although extensive research has identified associations between microbial composition and health outcomes, delineating direct causal mechanisms is constrained by high-dimensional datasets, potential confounders, and limitations in extrapolating findings from animal models to humans [390]. In this respect, several key barriers or challenges have been highlighted [391]. First, directionality of causality, commonly referred to as the “chicken-or-egg dilemma”: it remains unclear whether microbial communities in physiological or metabolic processes drive disease pathogenesis or arise secondary to disease-associated environmental alterations. Second, high dimensionality and compositionality, as microbiome datasets encompass thousands of taxa, make it challenging to determine whether disease phenotypes are attributable to specific species, groups of species, or the community as a whole. In addition, data are often compositional, expressed as relative abundances rather than absolute counts, which can generate spurious correlations. Third, inter-individual variability, since microbial profiles vary substantially between individuals, often exceeding disease-associated effects, complicating the identification of a universally causal “healthy” microbiome. Indeed, the high degree of inter-individual variability exhibited by the microbiome is a salient feature, with these differences potentially eclipsing the impact of its action. Fourth, limitations of model systems, as GF or conventional animal models do not fully replicate the human microbiome. Indeed, certain human microbes fail to colonize effectively in these models, and animals possess unique taxa absent in humans. Fifth, technical and methodological heterogeneity, in which variations in sample collection, storage, sequencing platforms, and analytical pipelines hinder cross-study comparability, contribute to significant reproducibility limitations. To overcome these barriers, current strategies increasingly include longitudinal study designs, sophisticated computational modeling, and validation using defined synthetic microbial communities [391].

In the context of the present review, it is pivotal to consider the inherent limitations of the studies included, as they can significantly influence the interpretation of their findings. First, many studies utilize a cross-sectional design and relatively small sample sizes, which limit statistical power and increase susceptibility to confounding factors. Second, most investigations of the GM have focused on genus- and phylum-level analyses, despite evidence that species-level differences are crucial for understanding functional variations within the GM. Third, the predominant use of 16S rRNA gene sequencing restricts taxonomic resolution and overlooks other key microbial components, including protozoa, viruses, and fungi. Fourth, current bacterial sequencing approaches often cannot reliably distinguish between beneficial and pathogenic taxa, underscoring the anticipated advantages of whole-genome metagenomics, such as shotgun sequencing, in future research. Fifth, a substantial portion of the evidence comes from preclinical models, which may not fully reflect human developmental complexity and idiosyncrasy. Sixth, although GM dysbiosis has been associated with NDDs such as ASD and ADHD, it remains unclear whether microbial alterations are causal or consequential. Seventh, early-life microbiome composition is influenced by multiple confounding factors, including mode of delivery, breastfeeding, antibiotic exposure, socioeconomic status, and host genetics, making it difficult to isolate the effects of individual bacterial species on neurodevelopment and immune function [8,392,393,394].

A further limitation in the current literature on the GBA and its role in developmental biology is the lack of precise characterization of microbial metabolite and neurotransmitter activity, including dosage, concentration specificity, temporal and spatial dynamics, and upstream and downstream signaling pathways. This gap has promoted a shift from purely observational studies toward mechanistic, causal investigations, including: (i) mapping 4-ethylphenyl sulfate (4EPS) to specific bacterial enzymes to assess its effects on oligodendrocyte maturation [395]; (ii) using bioengineered microorganisms to selectively produce metabolites and evaluate their impact on behavior [396,397]; and (iii) determining the exact molar concentrations of SCFAs required to inhibit histone deacetylases (HDACs) in specific human immune cell populations [398]. Taken together, all these core limitations, including methodological constraints, variability across individuals, differences between animal and human models, environmental and host-related confounders, and ethical restrictions in pediatric interventions (e.g., invasive sampling, long-term exposure risks, requirements for parental consent and child assent), underscore that the mechanistic understanding of GM-mediated brain and immune development remains incomplete. Addressing these challenges will require carefully designed human studies integrating multi-omics and functional assessments.

### 9.3. Limitations of the Review

The present review possesses several important limitations that should be acknowledged. First, as a narrative review, no formal risk-of-bias assessment or quantitative synthesis was performed. Second, much of the summarized evidence is predominantly correlational, limiting the ability to establish causality between GM alterations and neurodevelopmental or immune outcomes. Third, the review relies heavily on preclinical research, which may not fully translate to human physiology due to specific differences in GM composition, immune system development, and neurodevelopmental processes. Fourth, the scope of the review is very broad, which in some sections results in descriptive reporting rather than critical synthesis. Fifth, inconsistencies across studies, arising from differences in study design, population characteristics, sequencing platforms, taxonomic resolution, sample sizes, and analytic methodologies, complicate the derivation of definitive conclusions and limit generalizability. Sixth, mechanistic understanding remains incomplete, as many studies report associations without clarifying the temporal and spatial specificity of metabolites, signaling pathways, dose–response relationships, or upstream and downstream interactions, which constrains interpretation of functional relevance. Seventh, while the review discusses potential interventions, the current clinical evidence remains limited, heterogeneous, and not fully validated, preventing definitive recommendations regarding efficacy, safety, or population-specific applicability. Finally, methodological limitations, including the underrepresentation of longitudinal and multi-omics studies, insufficient consideration of confounding factors such as diet, antibiotics, delivery mode, breastfeeding, host genetics, and socioeconomic variables, as well as a lack of standardized protocols for microbial profiling, restrict the robustness and reproducibility of the conclusions. Consequently, interpretations of the findings must be approached with caution.

## 10. Conclusions

The GM plays a contributing role in shaping and modulating human physiological processes, including the development and function of both the brain and the immune system. Its influence appears to be primarily mediated through the synthesis of neurotransmitters and microbial metabolites, as well as through the activation of specific pathways within the HPA axis. However, the exact mechanisms through which the GM exerts these effects, and the full extent of its impact on neurodevelopmental and immune health, remain incompletely understood and continue to be active areas of research and scientific debate. Ultimately, advances revealing how the GM shapes early brain and immune system development will create new opportunities for innovative interventions and predictive strategies aimed at transforming pediatric health outcomes.

## Figures and Tables

**Table 1:** Major bacterial genera involved in the synthesis of various neurotransmitters.

Microbial Compounds	Bacterial Producers	Major Effects on the CNS	References
Serotonin (5-HT)	*Bifidobacterium*, *Lacticigenicum*, *Roseburia*	Increases vagus neuron activity and regulates both astrocyte and microglia activation.	[113,114]
Gamma-aminobutyric acid (GABA)	*Alistipes*, *Bacteroides*, *Bifidobacterium*, *Blautia*, *Escherichia*, *Lacticigenicum*, *Lactobacillus*	Promotes neuronal differentiation and regulates neuronal excitability.	[115,116]
Dopamine	*Bacillus*, *Bacteroides*, *Bifidobacterium*, *Brevilactibacter*	Regulates the intensity of synapses in neurons.	[117,118]
Norepinephrine	*Bacillus*, *Escherichia*, *Proteus*	Regulates synaptic plasticity, upregulates BDNF, and reduces pro-inflammatory factors.	[119]

CNS, central nervous system; BDNF, brain-derived neurotrophic factor.

**Table 2:** Pediatric diseases associated with alterations in GM composition and function.

Disease	GM Dysbiosis	Proposed Mechanisms	References
Type 1 diabetes mellitus	Decrease in SCFA-producers (*Faecalibacterium* and *Lachnospiraceae*). Increase in proinflammatory genera (*Prevotella*, *Streptococcus infantarius* and *Ruminococcus gnavus*).	Impaired SCFA production. Increased gut permeability. Loss of immune tolerance. β-cell autoimmunity.	[203,208,209]
Autism spectrum disorder	Decrease in *Prevotella*, *Veillonella*, and *Streptococcus.* Increase in *Candida.*	Innate immune dysregulation.	[205,210]
Inflammatory bowel disease	Decrease in SCFA-producers (*Faecalibacterium*, *Bacteroides* and *Roseburia*). Increase in *Enterobacteriaceae* and *Fusobacterium.*	Impaired SCFA-mediated immune regulation. Loss of barrier-protective and anti-inflammatory bacteria. Increased oxidative stress and inflammation.	[206,211,212]
Celiac disease	Decrease in *Streptococcus thermophilus*, *Faecalibacterium prausnitzii*, and *Clostridium clostridioforme*. Increase in *Dialister invisus*, *Parabacteroides*, and *Lachnospiraceae.*	Altered antigen processing. Increased inflammation. Impaired oral tolerance.	[213]
Juvenile idiopathic arthritis	Decrease in microbial diversity. Increase in *Veillonella* and *Collinsella.*	Altered gut permeability and translocation of microbial products. Autoimmune joint inflammation.	[214,215]

SCFA: short-chain fatty acids.

**Table 3:** Studies (2019–2025) on alterations in GM composition in ASD individuals.

Study/Country	Participants	Method	Increased Bacterial Taxa	Decreased Bacterial Taxa
Bundgaard-Nielsen et al. [258]/Denmark	*n* = 12 children and adolescents with ASD (5–17 years old)	16S V4 rRNA	*Eggerthella*, *Hungatella*, *Dielma*, *Ruminococus gnavus*	*Sutterella*, *Coprobacter*, *Paraprevotella*, *Bilophila*
Chang et al. [259]/Taiwan	*n* = 239 individuals with ASD (4–25 years old)	16S rRNA amplicon library preparation and Illumina V3V4	*Coprobacter*	*Blautia*, *Eubacterium hallii*, *Anaerostipes*, *Parasutterella*, *Erysipelotrichaceae UCG 003*, *Ruminococcaceae UCG 013*
Chen et al. [260]/China	*n* = 82 children and young with ASD (6–21 years old)	16S V3-V4 rRNA	*Fusobacterium*, *Ruminococcus torques*, *Bacteroides plebeius*	*Ruminococcaceae UCG 013*, *Erysipelotrichaceae UCG 003*, *Parasutterella*, *Clostridium* sensu stricto 1, *Turicibacter*, *Clostridium spiroforme*, *Intestinimonas butyriciproducens*
Chen et al. [261]/China	*n* = 138 children with ASD	16S rRNA	*Bacteroides*, *Faecalibacterium*	*Prevotella*
Dan et al. [262]/China	*n* = 143 children (2–13 years old)	16S rRNA	*Dialister*, *Escherichia*/*Shigella*, *Bifidobacterium*	*Prevotella 9*, *Megamonas*, *Ruminococcus 2*, *Sutterella*, *Prevotella*, *Bacteroides*
Ding et al. [263]/China	*n* = 25 children (mean age 5.7 years old)	16S V3-V4 rRNA	*Faecalibacterium*, *Prevotella*, *Subdoligranulum*, *Ruminococcus*	*Bifidobacterium*
Huang et al. [264]/China, USA, Taiwan	*n* = 39 children (3–6 years old)	16S rRNA amplicon library preparation and Illumina V4V5	*Escherichia*/*Shigella*, *Phyllobacterium*	NS
Liu et al. [265]/China	*n* = 30 children and young with ASD (2.5–18 years old)	16S rRNA	Members of the phylum Acidobacteriota	*Ruminococcaceae*, *Eubacterium*, *Lachnospiraceae*, *Erysipelotrichaceae*
Ma et al. [266]/China	*n* = 45 children with ASD (6–9 years old)	16S rRNA	*Megamonas*	*Acidaminococcaceae*, *Lachnoclostridium*, *Tyzzerella* subgroup 4, *Flavonifractor*, *Clostridium clostridioforme*
Niu et al. [267]/China	*n* = 114 children with ASD (3–8 years old)	Illumina MiSeq platform	*Lachnospira*	*Bacteroides*, *Bifidobacterium*, *Ruminococcus*, *Roseburia*, *Blautia*
Toscano de Oliveira et al. [268]/Brazil	*n* = 10 children with ASD (mean age 6.2 years old)	16S rRNA	*Clostridium* sensu stricto 1, *Ruminococcus torques*, *Lachnospiraceae UCG004*, *Bifidobacterium breve*	*Blautia*, *Bifidobacterium*
Wan et al. [269]/China	*n* = 64 children with ASD (3–6 years old)	Ilumina Novoseq 6000	*Clostridium*, *Dialister*, *Coprobacillus*, *Alistipes indistinctus*, *candidate TM7*, *Streptococcus cristatus*, *Streptococcus oligofermentans*, *Eubacterium limosum*	*Faecalibacterium*
Wang et al. [270]/China	*n* = 42 children with ASD (mean age 5.8 years old)	16S rRNA	*Streptococcus*, *Ruminococcus*, *Ruminiclostridium*	*Flavonifractor*, *Bradyrhizobium*
Zhai et al. [271]/China	*n* = 78 children with ASD (3–7 years old)	Illumina MiSeq platform	*Bacteroides*, *Parabacteroides*, *Sutterella*, *Lachnospira*, *Bacillus*, *Bilophila*, *Lactococcus*, *Lachnobacterium*, *Oscillospira*	NS
Zou et al. [272]/China	*n* = 48 children with ASD (2–7 years old)	Illumina MiSeq platform	*Bacteroides*, *Prevotella*, *Lachnospiracea incertae sedis*, *Megamonas*	*Clostridium XlVa*, *Eisenbergiella*, *Clostridium IV*, *Flavonifractor*, *Escherichia*/*Shigella*, *Haemophilus*, *Akkermansia*, *Dialister*
Zuffa et al. [273]/Sweden	*n* = 35 infants with ASD (5–36 months old)	Illumina NextSeq	*Clostridium neonatale*, *Klebsiella oxytoca*	*Bifidobacterium*

NS: not specified.

**Table 4:** Studies (2019–2025) on alterations in GM composition in ADHD individuals.

Study/Country	Participants	Method	Increased Bacterial Taxa	Decreased Bacterial Taxa
Bundgaard-Nielsen et al. [258]/Denmark	*n* = 59 children and adolescents with ADHD (5–17 years old)	16S V4 rRNA	*Streptococcus*, *Lactobacillus*, *Hungatella*, *Eggerthella*, *Ruminococcus gnavus*	*Coprobacter*, *Bilophila*, *Howardella*, *Colidextribacter*
Cassidy-Bushrow et al. [286]/USA	*n* = 59 infants with ADHD (2–10 years old)	16S rRNA	*Akkermansia*, *Blautia*, *Collinsella*, *Dorea*	*Enterococcus*, *Ruminococcus*
Lee et al. [292]/Taiwan	*n* = 54 children with ADHD (6–18 years old)	Illumina MiSeq platform	*Agathobacter*, *Prevotella 2*, *Phascolarctobacterium*, *Acidaminococcus*, *Roseburia*, *Ruminococcus gnavus*, *Parasutterella*	*Alistipes*, *Eubacterium eligens*, *Rikenellaceae*
Richarte et al. [293]/Spain	*n* = 100 adult medication-naïve ADHD subjects (18–59 years old)	16S V3-V4 rRNA	*Selenomonadaceae*, *Veillonellaceae*, *Dialister*, *Megamonas*	*Gracilibacteraceae*, *Anaerotaenia*, *Gracilibacter*
Steckler et al. [294]/Poland	*n* = 42 individuals with ADHD (6–18 years old)	Illumina MiSeq platform	*Blautia*, *Lachnospiraceae*	*Akkermansia*, *Anaerococcus*, *Christensenellaceae*, *Ruminococcaceae*
Szopinska-Tokov et al. [295]/The Netherlands	*n* = 42 individuals with ADHD (13–29 years old)	16S rRNA	*Ruminococcaceae UCG 003*, *Ruminococcaceae UCG 004*, *Ruminococcaceae UCG 005*, *Ruminiclostridium 9*, *Ruminococcus 2*	*Haemophilus*
Wan et al. [296]/China	*n* = 17 children with ADHD (6–12 years old)	Illumina NovaSeq platform	*Bacteroides caccae*, *Odoribacter splanchnicus*, *Paraprevotella xylaniphila*, *Veillonella párvula*, *Enterococcus*	*Faecalibacterium prausnitzii*, *Ruminococcus gnavus Lachnospiraceae*, *Veillonellaceae*
Wang et al. [288]/Taiwan	*n* = 30 children with ADHD (mean age 8.4 years old)	16S V3-V4 rRNA	*Bacteroides uniformis*, *B. ovatus*, *Sutterella stercoricanis*	*Bacteroides coprocola*
Wang et al. [297]/Taiwan	*n* = 41 children with ADHD (mean age 8 years old)	16S V3-V4 rRNA	*Agathobacter*, *Anaerostipes*, *Lachnospiraceae UCG-010*, *Roseburia*, *Ruminococcaceae*	*Prevotella 2*, *Prevotella 9*, *Scardovia*, *Phocaeicola plebeius*
Wang et al. [298]/China	*n* = 47 medication-naïve children and adolescents with ADHD (6–16 years old)	Metagenomic shotgun sequencing 2000 platform	*Lachnospiraceae*, *Lachnospira*, *Acidaminococcaceae*, *Phascolarctobacterium faecium*, *Anaerostipes hadrus*	*Odoribacter splanchnicus*, *Alistipes onderdonkii*, *A. shahii*, *Megamonas funiformis*, *Bacteroides caccae*, *Rikenellaceae*

**Table 5:** Studies (2019–2025) on alterations in GM composition in TD, TS, and CPE individuals.

Study/Country	Participants	Method	Increased Bacterial Taxa	Decreased Bacterial Taxa
Wang et al. [329]/China	*n* = 28 children with TD (6–14 years old)	16S rRNA	*Prevotella*, *Faecalibacterium*, *Coprobacillus*, *Odoribacter*	*Bifidobacterium*, *Collinsella*
Xi et al. [318]/China	*n* = 49 children with TD (mean age 8.84 years old)	Shotgun metagenomic sequencing	*Phocaeicola plebeius*, *Mediterraneibacter faecis*	*Prevotella stercorea*, *Streptococcus lutetiensis*
Bao et al. [330]/China	*n* = 32 children and preadolescents with TS (3–13 years old)	16S rDNA amplicon pyrosequencing	*Faecalibacterium*, *Hungatella*, *Oscillibacter*, *Flavonifractor*, *Fusicatenibacter*, *Anaerostipes*, *Anaerotruncus*, *Eisenbergiella*	*Clostridia UCG 014*
Huang et al. [323]/China	*n* = 25 children with CPE (3–18 years old)	Illumina MiSeq platform	*Bifidobacterium*, *Streptococcus*, *Akkermansia*, *Enterococcus*, *Prevotella*, *Veillonella*, *Rothia*, *Clostridium IV*	*Bacteroides*, *Faecalibacterium*, *Blautia*, *Ruminococcus*, *Roseburia*, *Anaerostipes*, *Parasutterella*
Peng et al. [326]/China	*n* = 13 children and preadolescents with CPE (1–16 years old)	Shotgun metagenomic sequencing	*Phascolarctobacterium faecium*, *Eubacterium limosum*	*Bacteroides fragilis*, *Dialister invisus*

## Data Availability

Not applicable.

## Abbreviations

The following abbreviations are used in this manuscript:

ACEs	Adverse childhood experiences
ADHD	Attention-deficit/hyperactivity disorder
AHR	Aryl hydrocarbon receptor
ANS	Autonomic nervous system
ASD	Autism spectrum disorder
BAs	Bile acids
BBB	Blood–brain barrier
BDNF	Brain-derived neurotrophic factor
CNS	Central nervous system
CPE	Cerebral palsy with epilepsy
CRF	Corticotropin-releasing factor
DG	Dentate gyrus
DMN	Default mode network
ENS	Enteric nervous system
FAS	Fetal alcohol syndrome
FASDs	Fetal alcohol spectrum disorders
FMT	Fecal microbiota transplantation
GABA	Gamma-aminobutyric acid
GBA	Gut–brain axis
GF	Germ-free
GI	Gastrointestinal
GM	Gut microbiome
GPRs	G-protein coupled receptors
HDACs	Histone deacetylases
HPA	Hypothalamic–pituitary–adrenal
5-HT	Serotonin
KYN	Kynurenine
LPS	Lipopolysaccharide
miRNAs	microRNAs
NDDs	Neurodevelopmental disorders
NMDA	N-methyl-D-aspartate
PAE	Prenatal alcohol exposure
PFC	Prefrontal cortex
PRRs	Pattern recognition receptors
ROI	Recurrence of illness index
RSFC	Resting state functional connectivity
SCFAs	Short-chain fatty acids
SGZ	Subgranular zone
SLs	Sphingolipids
SPF	Specific pathogen-free
SSRIs	Selective serotonin reuptake inhibitors
SVZ	Subventricular zone
TDs	Tic disorders
TLRs	Toll-like receptors
TPH1	Tryptophan hydroxylase type 1
TPH2	Tryptophan hydroxylase type 2
TS	Tourette syndrome
